# ELECTRA-STROKE: Electroencephalography controlled triage in the ambulance for acute ischemic stroke—Study protocol for a diagnostic trial

**DOI:** 10.3389/fneur.2022.1018493

**Published:** 2022-10-03

**Authors:** Maritta N. van Stigt, Anita A. G. A. van de Munckhof, Laura C. C. van Meenen, Eva A. Groenendijk, Monique Theunissen, Gaby Franschman, Martin D. Smeekes, Joffry A. F. van Grondelle, Geertje Geuzebroek, Arjen Siegers, Henk A. Marquering, Charles B. L. M. Majoie, Yvo B. W. E. M. Roos, Johannes H. T. M. Koelman, Wouter V. Potters, Jonathan M. Coutinho

**Affiliations:** ^1^Department of Clinical Neurophysiology, Amsterdam University Medical Centers (UMC) Location University of Amsterdam, Amsterdam, Netherlands; ^2^Department of Neurology, Amsterdam UMC Location University of Amsterdam, Amsterdam, Netherlands; ^3^Witte Kruis Ambulancezorg, Alkmaar, Netherlands; ^4^Ambulancezorg Nederland, Zwolle, Netherlands; ^5^Ambulance Amsterdam, Amsterdam, Netherlands; ^6^Department of Biomedical Engineering and Physics, Amsterdam UMC Location University of Amsterdam, Amsterdam, Netherlands; ^7^Department of Radiology and Nuclear Medicine, Amsterdam UMC Location University of Amsterdam, Amsterdam, Netherlands

**Keywords:** EEG, diagnostic method, prehospital triage, acute ischemic stroke, large vessel occlusion

## Abstract

**Background:**

Endovascular thrombectomy (EVT) is the standard treatment for large vessel occlusion stroke of the anterior circulation (LVO-a stroke). Approximately half of EVT-eligible patients are initially presented to hospitals that do not offer EVT. Subsequent inter-hospital transfer delays treatment, which negatively affects patients' prognosis. Prehospital identification of patients with LVO-a stroke would allow direct transportation of these patients to an EVT-capable center. Electroencephalography (EEG) may be suitable for this purpose because of its sensitivity to cerebral ischemia. The hypothesis of ELECTRA-STROKE is that dry electrode EEG is feasible for prehospital detection of LVO-a stroke.

**Methods:**

ELECTRA-STROKE is an investigator-initiated, diagnostic study. EEG recordings will be performed in patients with a suspected stroke in the ambulance. The primary endpoint is the diagnostic accuracy of the theta/alpha ratio for the diagnosis of LVO-a stroke, expressed by the area under the receiver operating characteristic (ROC) curve. EEG recordings will be performed in 386 patients.

**Discussion:**

If EEG can be used to identify LVO-a stroke patients with sufficiently high diagnostic accuracy, it may enable direct routing of these patients to an EVT-capable center, thereby reducing time-to-treatment and improving patient outcomes.

**Clinical trial registration:**

ClinicalTrials.gov, identifier: NCT03699397.

## Introduction

Stroke is the world's second leading cause of death and the third leading cause of death and disability combined (1). For more than two decades, intravenous thrombolysis (IVT) has been the standard treatment for acute ischemic stroke (AIS) (2). In 2015, various randomized controlled trials established the efficacy of endovascular thrombectomy (EVT) in patients with large vessel occlusion stroke of the anterior circulation (LVO-a stroke) (3), and EVT has since become standard therapy for this population (4, 5). For both IVT and EVT, early initiation of treatment is of the utmost importance, as patient outcome reduces with increasing time-to-treatment (6).

While IVT is available in most hospitals, EVT is—because of its complexity and required resources—only performed in selected hospitals, so-called comprehensive stroke centers (CSCs). Currently, ~45–55% of EVT-eligible patients are initially referred to primary stroke centers (PSCs), which are hospitals that do not provide this therapy (7–9). After initial workup, patients with LVO-a stroke must be transferred to a CSC. This workflow substantially delays the initiation of EVT, which has a negative effect on patient outcomes (6, 10). A study in the Netherlands found that the initial presentation of patients with LVO-a stroke in a PSC resulted in a delay of EVT by 1 h on average which was associated with an absolute decrease in the chance of functional independence at 3 months by 8.5% (7, 11). In other countries, the delay of EVT varied between 40 and 115 min in patients transferred to a CSC when compared with patients who were directly presented to a CSC (9, 12–14).

Since only a small proportion, ~12%, of all patients with a suspected stroke is eligible for EVT (15), it is not feasible to transport all patients with suspected stroke directly to a CSC. This would not only challenge the capacity of ambulance services and CSCs, but it would also lead to an unnecessary delay in the initiation of therapy in patients who require IVT but not EVT, as these patients would be subjected to a longer travel time.

**Figure 1:**
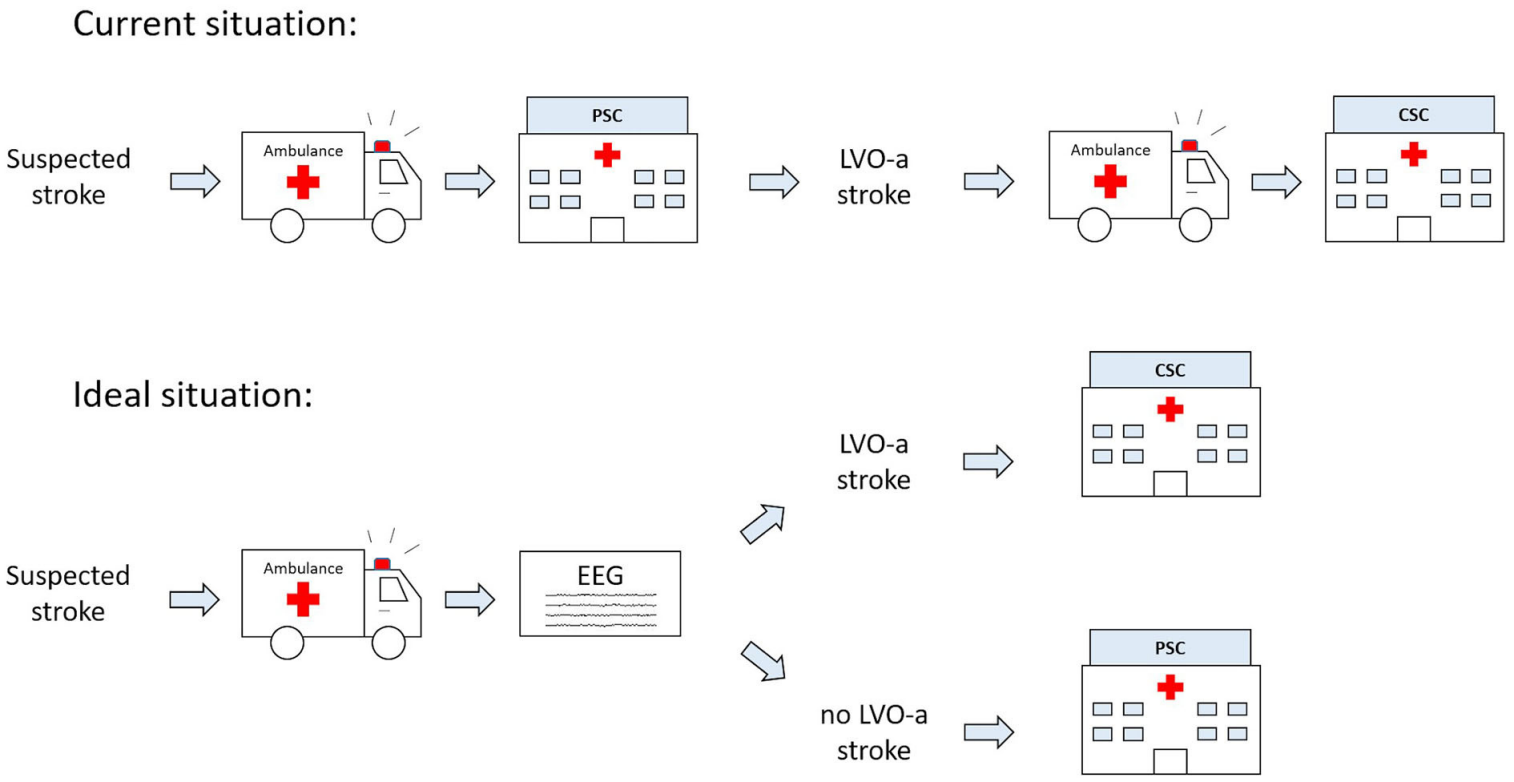
Current and future ideal prehospital workflow for patients with suspected acute ischemic stroke (AIS). CSC, comprehensive stroke center; EEG, electroencephalography; LVO-a, large vessel occlusion of the anterior circulation; PSC, primary stroke center.

A triage method that reliably identifies patients with LVO-a stroke in the ambulance would allow for patients with LVO-a stroke to be directly transported to a CSC, while patients without LVO-a stroke could still be transported to the nearest PSC (Figure 1). Several triage methods have been proposed for this purpose, but none of these methods are currently considered suitable for broad implementation (16). For example, multiple studies on the use of Mobile Stroke Units for prehospital diagnosis and treatment of acute ischemic stroke have been conducted. These studies show a positive effect of Mobile Stroke Units on the functional outcomes of patients (17–19). However, the costs of Mobile Stroke Units are high (20, 21), which makes broad implementation difficult.

Electroencephalography (EEG) may be suitable as a prehospital triage method since EEG is highly sensitive to changes in neuronal function caused by brain ischemia (22). Previous studies have shown that a decrease in cerebral blood flow results in a slowing of the EEG signal within seconds (23, 24). Multiple case-control studies have found that ratios between slow and fast EEG activity, such as the delta/alpha ratio, discriminate between patients with AIS and healthy controls (25, 26). Few studies have been conducted on the use of EEG to identify LVO stroke in patients with suspected acute ischemic stroke (27, 28). A study used a portable LVO detection device using EEG and somatosensory-evoked potentials and demonstrated high LVO discrimination (C-statistic: 0.88) (29). Another study used a dry electrode EEG system and reported a high diagnostic accuracy for LVO stroke detection when combining clinical and EEG data [area under the receiver operating characteristic (ROC) curve: 87.8] (30). All these studies, however, were conducted in the emergency room and not in the prehospital setting.

We hypothesize that EEG accurately identifies the presence of an LVO-a stroke in patients with a suspected stroke when applied in the ambulance. The aim of the ELECTRA-STROKE study is to determine the diagnostic accuracy of dry electrode cap EEG for the diagnosis of LVO-a stroke when performed by ambulance personnel in patients with a suspected stroke.

## Methods and analysis

### Study design and patient population

The ELECTRA-STROKE study is an investigator-initiated, diagnostic, multi-center study consisting of four phases. In this study protocol article, we describe the fourth and final phase, which is currently being carried out by two ambulance organizations in the Netherlands (Ambulance Amsterdam and Witte Kruis Ambulancezorg Alkmaar). After a 6-month delay due to COVID-19, patient enrollment started in August 2020. We are enrolling patients who are referred to one of the participating hospitals, namely, Amsterdam University Medical Centers (UMC), OLVG hospitals, or Noordwest Ziekenhuisgroep. Inclusion criteria are clinically suspected stroke as determined at the discretion of ambulance personnel, an age of 18 years or older, and an onset of symptoms or time last seen well <24 h before the start of the EEG recording. Patients who are diagnosed with LVO-a stroke in a PSC and who are transferred to a CSC for EVT treatment are eligible for inclusion at the time of inter-hospital transfer. Injury or active infection of electrode cap placement area, and (suspected) COVID-19 infection are exclusion criteria for the study.

### Study procedures

**Figure 2:**
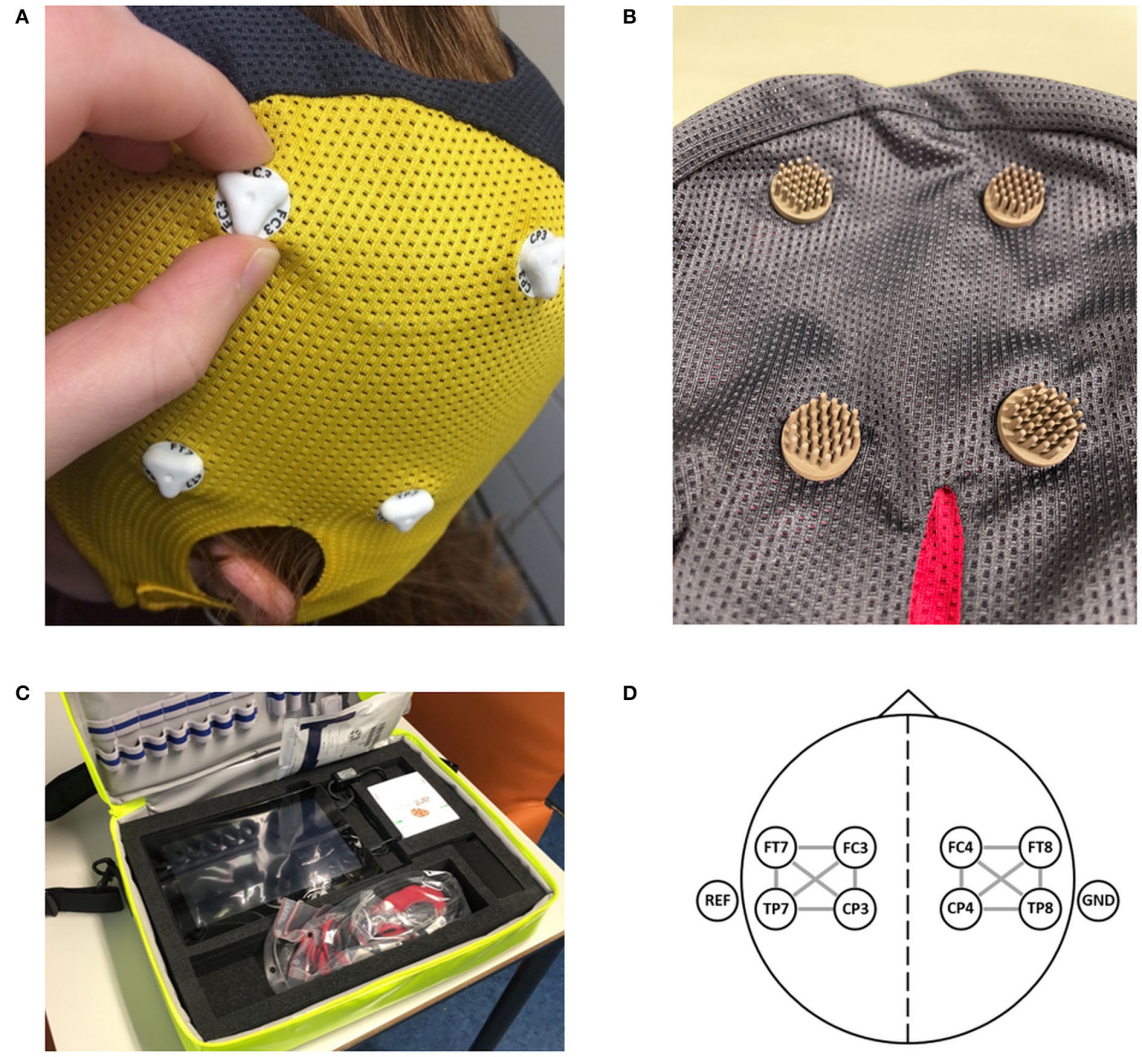
The equipment as used in the ELECTRA-STROKE study. **(A)** The 8-channel dry electrode cap (Waveguard touch, Eemagine, Berlin, Germany). **(B)** The multipin Ag/AgCl coated dry electrode. **(C)** The portable and lightweight EEG equipment, including the dry electrode EEG cap (weight ±50 g) and EEG amplifier (eegoTM amplifier EE-411, Eemagine, Berlin, Germany). **(D)** Electrode layout with 12 bilateral bipolar channels. Figures 2A,D have previously been published [van Meenen LCC, van Stigt MN, Marquering HA, et al. Detection of large vessel occlusion stroke with electroencephalography in the emergency room: first results of the ELECTRA-STROKE study. *J Neurol*. 2022;269(4):2030–2038. doi: 10.1007/s00415-021-10781-6; https://creativecommons.org/licenses/by/4.0/].

**Figure 3:**
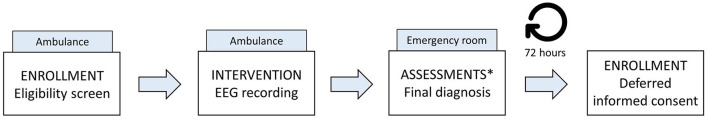
Flowchart of enrollment, intervention, and assessments in the study. There is no follow-up. *Assessments include patient characteristics, such as demographics, medical history, medication use, and data obtained at the emergency room as part of the work-up for the suspected acute ischemic stroke, such as the neurological examination, radiological data, final diagnosis, and treatment.

We will collect a single dry electrode cap EEG recording per patient as well as clinical data and imaging data. The EEG recordings are performed by ambulance personnel using dry electrode EEG caps with eight electrodes in positions FC3, FC4, CP3, CP4, FT7, FT8, TP7, and TP8 (Waveguard touch, Eemagine, Berlin, Germany; Figures 2A,B), and compatible EEG amplifiers (eego amplifier EE-411, Eemagine, Berlin, Germany). EEG data are acquired using NeuroCenter^®^ EEG software (Clinical Science Systems, Leiden, The Netherlands). Ambulance personnel attend a 1-h training course in performing dry electrode EEG recordings for the purpose of this study. All equipment is contained in a portable and lightweight bag, specifically designed for use by ambulance personnel (Figure 2C). We instruct ambulance personnel to record the EEG data while the patient is lying on the ambulance stretcher with their head resting on the headrest to minimalize EEG artifacts due to movement and muscle activity. EEG recordings are performed either at the patient's site or in the ambulance before departure to the hospital. To minimize any delay, automatic time limits were added to both the electrode positioning step (1.5 min) and the EEG recording step (2.5–3.0 min) in the acquisition software. Acquired EEG data are electronically sent to the research team to be analyzed retrospectively. After the EEG data are acquired, ambulance personnel follow local protocols for the workup of patients with suspected stroke. A flowchart of the enrollment, intervention, and assessments of enrolled patients is provided in Figure 3

Clinical data are retrieved from the medical records. Patient characteristics, such as demographics, medical history, and medication use are collected. In addition, we collect data obtained at the emergency room as part of the workup for the suspected stroke, such as the neurological examination, radiological data, final diagnosis, and treatment. All clinical study data are stored in coded form in an online secured database (Castor EDC) (31). EEG data are also coded and stored on a local secured drive. The identification key is secured by a password and stored on a network drive of the coordinating institution. Only the research team of the coordinating institution has access to these data. More detailed information about the data collection and management procedures can be found in paragraphs 8.3 and 12.1 of the research protocol (Supplementary material 1). There are no planned follow-up visits. Patient inclusion will continue until the number of required patients, according to the sample size calculation, has been reached.

### Definitions and outcomes

The primary endpoint of the study is the diagnostic accuracy of dry electrode cap EEG to discriminate LVO-a stroke from all other strokes and stroke mimics in the prehospital setting, expressed as the area under the ROC curve of the theta/alpha ratio. The theta/alpha ratio is normalized between −1 and 1 using the following equation:


$$\begin{array}{l} TAR=\;\frac{P\left(\theta \right)-P\left(\alpha \right)}{P\left(\theta \right)+P\left(\alpha \right)} \end{array}$$

where *P*(θ) is the power in the theta frequency band (4–8 Hz) and *P*(α) is the power in the alpha frequency band (8–12 Hz). The sensitivity, specificity, positive predictive value, and negative predictive value will also be determined. The theta/alpha ratio was chosen as the EEG feature for the primary endpoint based on the results of a previously published study in which EEG was evaluated in patients with stroke in the hospital setting (28).

An LVO-a stroke is defined as an occlusion of the intracranial part of the internal carotid artery, the M1 or proximal M2 segment of the middle cerebral artery, or the A1 segment of the anterior cerebral artery. The occlusion location is determined by an adjudication committee based on computed tomography (CT) angiography imaging. In case a patient did not undergo CT angiography, the patient is scored as having no LVO-a stroke.

The secondary endpoints are the diagnostic accuracies of the following EEG features: relative delta, theta, alpha, and beta power, the delta/alpha ratio, delta+theta/alpha+beta ratio, pairwise derived brain symmetry index, and weighted phase lag index. Further outcomes are the logistical and technical feasibility of ambulance personnel performing an EEG recording with a dry electrode cap in the prehospital setting in patients with suspected stroke, and the development of a novel EEG-based algorithm for the detection of LVO-a stroke.

The principal safety outcome is the occurrence of device-related adverse events. All adverse events that occurred within 1 h after the completion of the EEG recording, reported spontaneously by the subject or observed by the investigator, will be recorded. All adverse events will be followed until they have abated, or until a stable situation has been reached. This study is considered a low-risk study since EEG is a safe, non-invasive, and painless procedure that is used regularly in standard medical practice, and we are using CE-marked products only.

### Sample size

The sample size is calculated based on an expected specificity of dry electrode cap EEG for LVO-a stroke detection of 70%. Originally, the sample size was set at 222, based on an expected incidence of LVO-a stroke of 7% and a dropout of 20%. However, an interim analysis, based on 137 suspected stroke patients in whom a dry electrode cap EEG recording was performed in the prehospital setting, showed an actual incidence of LVO-a stroke of 5% and a dropout rate of 79/137 (58%), mainly caused by insufficient EEG data quality [72/137 (53%)]. Patients were considered a dropout if EEG data were of insufficient quality, the onset of symptoms was >24 h before the start of the EEG recording, or no informed consent was provided. For future EEG recordings, we expect a lower dropout rate as continuous software and hardware improvements as well as increasing EEG experience of the users are expected to result in increased EEG data quality over the course of the study. Based on the revised estimations of LVO-a stroke proportion and drop-out rate, the sample size was recalculated. Assuming an incidence of LVO-a stroke of 5% among patients with a suspected stroke in the prehospital setting, an alpha of 0.05 and a maximum margin of error of 7% indicate that 174 patients are required (32). Assuming a dropout rate of 55%, we plan to perform EEG recordings in 386 patients with a suspected stroke or known LVO-a stroke in the prehospital setting. If multiple EEG recordings are performed in the same patients, we will only use the first EEG recording.

### Data analysis

EEG data will be re-referenced to a 12-channel bipolar montage with 6 bipolar derivations located at each hemisphere (Figure 2D). Then, artifacts will be detected and rejected. For each hemisphere, we will calculate the relative delta power, theta power, alpha power, beta power, the delta/alpha ratio, theta/alpha ratio, the delta+theta/alpha+beta ratio, and the weighted phase lag index (33). The features of the affected hemisphere of patients with LVO-a stroke will be compared to the features of the affected hemisphere of patients with a stroke or transient ischemic attack and the features of both hemispheres (if available) of patients with a stroke mimic. As a measure of brain symmetry, we will determine the pairwise derived brain symmetry index (34), using bipolar derivations located symmetrically on both hemispheres. We will evaluate the diagnostic accuracy of individual features by calculating the ROC curves and the area under these curves. Optimal cut-off values will be determined as the highest sensitivity at a specificity of ≥ 70% and ≥ 80% for LVO-a stroke. For these cut-off values, the sensitivity, specificity, negative predictive value, and positive predictive value will be reported with 95% confidence intervals.

Baseline characteristics will be compared between patients with LVO-a stroke and those without LVO-a stroke using the independent samples *t*-test for normally distributed continuous variables, the Mann–Whitney *U*-test for non-normally distributed continuous variables, and the chi-square test for categorical variables. Fisher's exact test will be used for binary categorical variables in case cells have an expected count of <5. Data will be analyzed using Python (Python Software Foundation) and are not visually interpreted by a neurologist.

#### Logistical and technical feasibility

To determine whether performing dry electrode cap EEG recordings is feasible in the prehospital stroke setting, we will assess the EEG data quality. Data with no or minor artifacts will be considered as having sufficient quality for further analysis. We will quantify the technical feasibility by calculating the percentage of patients with EEG data of sufficient quality for analysis, and the percentage of EEG data of sufficient quality for analysis. We will analyze the data over time to account for learning effects and hard- and software improvements.

#### Algorithm development

We intend to use EEG data to develop a logistic regression algorithm for LVO-a stroke prediction. We will calculate the EEG features specified in Supplementary Table 1 as possible inputs for the classification algorithm. These features will be averaged per hemisphere, except for the pairwise derived brain symmetry index, which will be calculated using bipolar derivations on both hemispheres resulting in one value per subject. Missing EEG data will be imputed. The number of input EEG features will thereafter be reduced to account for collinearity. Regularization will be used to further prevent the overfitting of the models. The diagnostic accuracy of the developed classification algorithm will be assessed by calculating ROC curves.

## Discussion

The aim of the ELECTRA-STROKE study is to evaluate the diagnostic accuracy of dry electrode cap EEG for the detection of LVO-a stroke, and the feasibility of ambulance personnel performing EEG recordings in patients with suspected stroke in the prehospital setting. If EEG proves to have both high sensitivity and specificity for LVO-a stroke, this would allow for direct transfer of patients with LVO-a stroke to a CSC in the future, while the other patients with suspected stroke will still be presented at the closest hospital where, if indicated, IVT can be initiated. This would save precious time for patients with LVO-a stroke and thereby increase the chance of good functional outcomes for these patients.

If EEG were to be used as a prehospital stroke triage instrument, it should not only have high diagnostic accuracy but must also be fast and easy to apply (16). A traditional wet electrode EEG does not meet these requirements as it requires time-consuming preparation as well as extensive training in correct electrode placement and optimization of the electrode-skin impedance (35). A dry electrode EEG cap as used in this study, however, overcomes these issues. Dry electrodes are designed to penetrate the hair layer and make direct contact with the scalp, without skin preparation (36, 37). Furthermore, since the electrodes are integrated into a cap, they are easy to apply without the need for extensive training (28). Together with a maximum EEG recording time of 2.5 min in the ambulance, and automated analysis of the EEG data by, e.g., an artificial intelligence-based algorithm, we expect the total time needed for an EEG-based diagnosis to be below 5 min. Although this would slightly delay the work-up of patients with non-LVO-a stroke, it would improve the time-to-treatment for patients with LVO-a stroke significantly (in the Netherlands by ~1 h). The time loss caused by an EEG-based diagnosis should, however, be limited to minimalize the delay in presentation of and, if applicable, treatment for patients with non-LVO-a stroke.

A challenge in using dry electrode EEG in the prehospital stroke setting is achieving good EEG data quality. Although some studies report similar data quality of dry electrode EEG compared with wet electrode EEG, there are also studies that show an increase in power in the lower frequencies (<3 Hz) for dry electrode EEG (37, 38). We should therefore interpret the results regarding the delta frequency band with care. Additionally, dry electrode EEG—when performed in a stable setting—has relatively low channel reliability of ~80% compared to wet electrodes (97%) (39). We expect that the channel reliability of dry electrode EEG—when performed in the acute stroke setting—will be even lower. Therefore, we will probably not be able to use the EEG data of all eight channels in our analysis.

## Ethics and dissemination

### Ethical approval

Ethical approval for this study has been obtained by the medical ethics review committee of Amsterdam UMC (reference number: 2018_175).

### Informed consent

Written informed consent will be obtained from all patients or their legal representatives. Due to the emergency setting, the ethical review board approved the use of a deferred consent procedure in compliance with the Declaration of Helsinki and Dutch law. Informed consent will be obtained by the local investigator as soon as feasible after EEG recording, preferably within 72 h after arrival at the hospital. If deferred consent cannot be obtained, all previously collected data (including the EEG recording) will be destroyed, and the patient will be excluded. Subjects can leave the study at any time for any reason if they wish to do so without any consequences.

## Conclusion

ELECTRA-STROKE is a diagnostic study that aims to evaluate the diagnostic accuracy of dry electrode cap EEG for the detection of LVO-a stroke in the prehospital setting. If EEG can identify LVO-a stroke patients with high diagnostic accuracy in the prehospital setting, it may enable direct routing of these patients to a CSC, thereby reducing time-to-treatment and improving patient outcomes.

## Trial status

The first patient was enrolled in August 2020. Currently, 321 patients have been included in this study. Patient recruitment is expected to be completed in September 2022.

## Ethics statement

The study involving human participants were reviewed and approved by the medical review committee of Amsterdam UMC. The patients/participants provided their written informed consent to participate in this study.

## Author contributions

JC, WP, LM, MvS, AM, and EG conceived the study, were involved in protocol development, and gaining ethical approval. MvS and AM wrote the first draft of the manuscript. All authors reviewed the manuscript and approved the final version of the manuscript.

## Funding

This work was supported by the Dutch Heart Foundation (2018T001), Health~Holland, and an unrestricted research grant from Medtronic. These funding sources had no role in the design of this study, its execution, analyses, interpretation of the data, and decision to submit results.

## Conflict of interest

Author CM reports grants from the CVON/Dutch Heart Foundation, European Commission, TWIN Foundation, Stryker, Healthcare Evaluation Netherlands, all outside the submitted work (paid to institution), and is a shareholder of Nico.lab, a company that focuses on the use of artificial intelligence for medical image analysis. Author YR is a minor shareholder of Nico.lab. Author HM is co-founder and shareholder of Nico.lab and Trianect, a startup that focuses on prehospital stroke care. Author JC received related research support from the Dutch Heart Foundation, Medtronic, unrelated research support from Bayer and Boehringer (all fees were paid to his employer), and is co-founder and shareholder of Trianect. Author WP received related research support from the Dutch Heart Foundation and is a co-founder and shareholder of Trianect. Authors MT and GF were employed by Witte Kruis Ambulancezorg. Author MS was employed by Ambulancezorg Nederland. Authors JG, GG, and AS were employed by Ambulance Amsterdam. The remaining authors declare that the research was conducted in the absence of any commercial or financial relationships that could be construed as a potential conflict of interest.

## Publisher's note

All claims expressed in this article are solely those of the authors and do not necessarily represent those of their affiliated organizations, or those of the publisher, the editors and the reviewers. Any product that may be evaluated in this article, or claim that may be made by its manufacturer, is not guaranteed or endorsed by the publisher.

## References

[1] GBD 2016 Stroke Collaborators. Global, Regional, and National Burden of Stroke and Its Risk Factors, 1990–2019: a systematic analysis for the Global Burden of Disease Study 2019. Lancet Neurol. (2021) 20:795–820. 10.1016/S1474-4422(21)00252-0 34487721 PMC8443449

[2] The National Institute of Neurological Disorders and Stroke rt-PA Stroke Study Group. Tissue plasminogen activator for acute ischemic stroke. N Engl J Med. (1995) 333:1581–7. 10.1056/NEJM199512143332401 7477192

[3] Goyal M Menon BK van Zwam WH Dippel DW Mitchell PJ Demchuk AM . Endovascular thrombectomy after large-vessel ischaemic stroke: a meta-analysis of individual patient data from five randomised trials. Lancet. (2016) 387:1723–31. 10.1016/S0140-6736(16)00163-X 26898852

[4] Albers GW Marks MP Kemp S Christensen S Tsai JP Ortega-Gutierrez S . Thrombectomy for stroke at 6 to 16 hours with selection by perfusion imaging. N Engl J Med. (2018) 378:708–18. 10.1056/NEJMoa1713973 29364767 PMC6590673

[5] Nogueira RG Jadhav AP Haussen DC Bonafe A Budzik RF Bhuva P . Thrombectomy 6 to 24 hours after stroke with a mismatch between deficit and infarct. N Engl J Med. (2018) 378:11–21. 10.1056/NEJMoa1706442 29129157

[6] Saver JL Goyal M van der Lugt A Menon BK Majoie CB Dippel DW . Time to treatment with endovascular thrombectomy and outcomes from ischemic stroke: a meta-analysis. J Am Med Assoc. (2016) 316:1279–88. 10.1001/jama.2016.13647 27673305

[7] Venema E Groot AE Lingsma HF Hinsenveld W Treurniet KM Chalos V . Effect of interhospital transfer on endovascular treatment for acute ischemic stroke. Stroke. (2019) 50:923–30. 10.1161/STROKEAHA.118.024091 30862265 PMC6430601

[8] Farouil G Sablot D Leibinger F Van Damme L Coll F Gaillard N . Mechanical recanalization after transfer from a distant primary stroke center: effectiveness and future directions. J Stroke Cerebrovasc Dis. (2019) 28:104368. 10.1016/j.jstrokecerebrovasdis.2019.104368 31537417

[9] Froehler MT Saver JL Zaidat OO Jahan R Aziz-Sultan MA Klucznik RP . Interhospital transfer before thrombectomy is associated with delayed treatment and worse outcome in the stratis registry (systematic evaluation of patients treated with neurothrombectomy devices for acute ischemic stroke). Circulation. (2017) 136:2311–21. 10.1161/CIRCULATIONAHA.117.028920 28943516 PMC5732640

[10] van Meenen LCC Riedijk F Stolp J van der Veen B Halkes PHA van der Ree TC . Pre- and interhospital workflow times for patients with large vessel occlusion stroke transferred for endovasvular thrombectomy. Front Neurol. (2021) 12:730250. 10.3389/fneur.2021.730250 34512538 PMC8428365

[11] van Meenen LCC Groot AE Venema E Emmer BJ Smeekes MD Kommer GJ . Interhospital transfer vs. direct presentation of patients with a large vessel occlusion not eligible for iv thrombolysis. J Neurol. (2020) 267:2142–50. 10.1007/s00415-020-09812-5 32266543 PMC7320925

[12] Jayaraman MV Hemendinger ML Baird GL Yaghi S Cutting S Saad A . Field triage for endovascular stroke therapy: a population-based comparison. J Neurointerv Surg. (2020) 12:233–9. 10.1136/neurintsurg-2019-015033 31484698

[13] Edwards LS Blair C Cordato D McDougall A Manning N Cheung A . Impact of interhospital transfer on patients undergoing endovascular thrombectomy for acute ischaemic stroke in an Australian setting. BMJ Neurol Open. (2020) 2:e000030. 10.1136/bmjno-2019-000030 33681779 PMC7903172

[14] Taschner CA Trinks A Bardutzky J Brich J Hartmann R Urbach H . Drip-and-ship for thrombectomy treatment in patients with acute ischemic stroke leads to inferior clinical outcomes in a stroke network covering vast rural areas compared to direct admission to a comprehensive stroke center. Front Neurol. (2021) 12:743151. 10.3389/fneur.2021.743151 34790162 PMC8591070

[15] Duvekot MHC Venema E Rozeman AD Moudrous W Vermeij FH Biekart M . Comparison of eight prehospital stroke scales to detect intracranial large-vessel occlusion in suspected stroke (presto): a prospective observational study. Lancet Neurol. (2021) 20:213–21. 10.1016/S1474-4422(20)30439-7 33422191

[16] van Meenen LCC van Stigt MN Siegers A Smeekes MD van Grondelle JAF Geuzebroek G . Detection of large vessel occlusion stroke in the prehospital setting: electroencephalography as a potential triage instrument. Stroke. (2021) 52:e347–55. 10.1161/STROKEAHA.120.033053 33940955

[17] Ebinger M Siegerink B Kunz A Wendt M Weber JE Schwabauer E . Association between dispatch of mobile stroke units and functional outcomes among patients with acute ischemic stroke in Berlin. J Am Med Assoc. (2021) 325:454–66. 10.1001/jama.2020.26345 33528537 PMC7856548

[18] Zhou T Zhu L Wang M Li T Li Y Pei Q . Application of mobile stroke unit in prehospital thrombolysis of acute stroke: experience from China. Cerebrovasc Dis. (2021) 50:520–5. 10.1159/000514370 34175842

[19] Grotta JC Yamal JM Parker SA Rajan SS Gonzales NR Jones WJ . Prospective, multicenter, controlled trial of mobile stroke units. N Engl J Med. (2021) 385:971–81. 10.1056/NEJMoa2103879 34496173

[20] Chen J Lin X Cai Y Huang R Yang S Zhang G . Systematic review of mobile stroke unit among acute stroke patients: time metrics, adverse events, functional result and cost-effectiveness. Front Neurol. (2022) 13:803162. 10.3389/fneur.2022.803162 35356455 PMC8959845

[21] Kim J Easton D Zhao H Coote S Sookram G Smith K . Economic evaluation of the melbourne mobile stroke unit. Int J Stroke. (2021) 16:466–75. 10.1177/1747493020929944 32536328

[22] van Putten MJ Hofmeijer J. EEG monitoring in cerebral ischemia: basic concepts and clinical applications. J Clin Neurophysiol. (2016) 33:203–10. 10.1097/WNP.0000000000000272 27258443

[23] Sundt TM Jr Sharbrough FW Anderson RE Michenfelder JD. Cerebral blood flow measurements and electroencephalograms during carotid endarterectomy. J Neurosurg. (1974) 41:310–20. 10.3171/jns.1974.41.3.0310 4412366

[24] Sharbrough FW Messick JM Jr Sundt TM Jr. Correlation of continuous electroencephalograms with cerebral blood flow measurements during carotid endarterectomy. Stroke. (1973) 4:674–83. 10.1161/01.STR.4.4.674 4723697

[25] Van Kaam RC van Putten M Vermeer SE Hofmeijer J. Contralesional brain activity in acute ischemic stroke. Cerebrovasc Dis. (2018) 45:85–92. 10.1159/000486535 29510399

[26] Finnigan S Wong A Read S. Defining abnormal slow EEG activity in acute ischaemic stroke: delta/alpha ratio as an optimal QEEG index. Clin Neurophysiol. (2016) 127:1452–9. 10.1016/j.clinph.2015.07.014 26251106

[27] Sutcliffe L Lumley H Shaw L Francis R Price CI. Surface electroencephalography (EEG) during the acute phase of stroke to assist with diagnosis and prediction of prognosis: a scoping review. BMC Emerg Med. (2022) 22:29. 10.1186/s12873-022-00585-w 35227206 PMC8883639

[28] van Meenen LCC van Stigt MN Marquering HA Majoie C Roos Y Koelman J . Detection of large vessel occlusion stroke with electroencephalography in the emergency room: first results of the electra-stroke study. J Neurol. (2022) 269:2030–8. 10.1007/s00415-021-10781-6 34476587 PMC8412867

[29] Sergot PB Maza AJ Derrick BJ Smith LM Berti LT Wilcox MR . Portable neuromonitoring device detects large vessel occlusion in suspected acute ischemic stroke. Stroke. (2021) 52:1437–40. 10.1161/STROKEAHA.120.031225 33596672

[30] Erani F Zolotova N Vanderschelden B Khoshab N Sarian H Nazarzai L . Electroencephalography might improve diagnosis of acute stroke and large vessel occlusion. Stroke. (2020) 51:3361–5. 10.1161/STROKEAHA.120.030150 32942967 PMC7606743

[31] Castor EDC,. Castor Electronic Data Capture. (2019). Available online at: https://castoredc.com (accessed August 28, 2019).

[32] Hajian-Tilaki K. Sample size estimation in diagnostic test studies of biomedical informatics. J Biomed Inform. (2014) 48:193–204. 10.1016/j.jbi.2014.02.013 24582925

[33] Vinck M Oostenveld R van Wingerden M Battaglia F Pennartz CM. An improved index of phase-synchronization for electrophysiological data in the presence of volume-conduction, noise and sample-size bias. Neuroimage. (2011) 55:1548–65. 10.1016/j.neuroimage.2011.01.055 21276857

[34] Sheorajpanday RV Nagels G Weeren AJ van Putten MJ De Deyn PP. Reproducibility and clinical relevance of quantitative EEG parameters in cerebral ischemia: a basic approach. Clin Neurophysiol. (2009) 120:845–55. 10.1016/j.clinph.2009.02.171 19375386

[35] Teplan M. Fundamental of EEG measurement. Measur Sci Rev. (2002) 2. Available online at: http://www.edumed.org.br/cursos/neurociencia/MethodsEEGMeasurement.pdf

[36] Fiedler P Griebel S Pedrosa P Fonseca C Vaz F Zentner L . Multichannel EEG with novel Ti/Tin dry electrodes. Sens Actuat. (2015) 221:139–47. 10.1016/j.sna.2014.10.010

[37] Fiedler P Pedrosa P Griebel S Fonseca C Vaz F Supriyanto E . Novel multipin electrode cap system for dry electroencephalography. Brain Topogr. (2015) 28:647–56. 10.1007/s10548-015-0435-5 25998854

[38] Fiedler P Pedrosa P Griebel S Fonseca C Vaz F Zanow F . Novel flexible dry Pu/Tin-multipin electrodes: first application in EEG measurements. Annu Int Conf IEEE Eng Med Biol Soc. (2011) 2011:55–8. 10.1109/IEMBS.2011.6089895 22254249

[39] di Fronso S Fiedler P Tamburro G Haueisen J Bertollo M Comani S. Dry EEG in sports sciences: a fast and reliable tool to assess individual alpha peak frequency changes induced by physical effort. Front Neurosci. (2019) 13:982. 10.3389/fnins.2019.00982 31619953 PMC6763587

