# Supplementary material for: ELECTRA-STROKE: Electroencephalography controlled triage in the ambulance for acute ischemic stroke—Study protocol for a diagnostic trial
Source: Front Neurol. 2022 Oct 3;13:1018493. doi: 10.3389/fneur.2022.1018493 (PMC9576201; doi:10.3389/fneur.2022.1018493)
Supplement: Supplementary file 1 [file Table_1.DOCX]

***SUPPLEMENTARY MATERIAL***

**Supplemental Table 1**. EEG features included in the analysis for algorithm development.

| **Group** | **EEG features** |
| --- | --- |
| Time domain |  |
| Data distribution(1) | Kurtosis  Skewness |
| Complexity(2, 3) | Sample entropy  Higuchi fractal dimension |
| Phase synchronization(4) | Weighted phase lag index  Magnitude squared coherence |
| Frequency domain |  |
| Frequency band power(5, 6) | Delta power (1-4 Hz)  Theta power (4-8 Hz)  Alpha power (8-13 Hz)  Beta power (13-18 Hz)  Delta/alpha power  Theta/alpha power  (Delta+theta)/(alpha+beta) power |
| Frequency asymmetry(7) | Pairwise derived brain symmetry index |

**Supplemental References**

1. Hussain I, Park, S.J. *Healthsos: Real-Time Health Monitoring System for Stroke Prognostics*. IEEE Access (2020). p. 213574-86.doi: 10.1109/ACCESS.2020.3040437.

2. Liu S, Guo J, Meng J, Wang Z, Yao Y, Yang J, et al. Abnormal Eeg Complexity and Functional Connectivity of Brain in Patients with Acute Thalamic Ischemic Stroke. *Comput Math Methods Med* (2016) 2016:2582478. Epub 2016/07/13. doi: 10.1155/2016/2582478.

3. Rubega M, Formaggio E, Molteni F, Guanziroli E, Di Marco R, Baracchini C, et al. Eeg Fractal Analysis Reflects Brain Impairment after Stroke. *Entropy (Basel)* (2021) 23(5). Epub 2021/06/03. doi: 10.3390/e23050592.

4. Van Kaam RC, van Putten M, Vermeer SE, Hofmeijer J. Contralesional Brain Activity in Acute Ischemic Stroke. *Cerebrovascular diseases (Basel, Switzerland)* (2018) 45(1-2):85-92. Epub 2018/03/07. doi: 10.1159/000486535.

5. Finnigan S, Wong A, Read S. Defining Abnormal Slow Eeg Activity in Acute Ischaemic Stroke: Delta/Alpha Ratio as an Optimal Qeeg Index. *Clinical neurophysiology : official journal of the International Federation of Clinical Neurophysiology* (2016) 127(2):1452-9. Epub 2015/08/08. doi: 10.1016/j.clinph.2015.07.014.

6. Shreve L, Kaur A, Vo C, Wu J, Cassidy JM, Nguyen A, et al. Electroencephalography Measures Are Useful for Identifying Large Acute Ischemic Stroke in the Emergency Department. *Journal of stroke and cerebrovascular diseases : the official journal of National Stroke Association* (2019) 28(8):2280-6. Epub 2019/06/09. doi: 10.1016/j.jstrokecerebrovasdis.2019.05.019.

7. Sheorajpanday RV, Nagels G, Weeren AJ, van Putten MJ, De Deyn PP. Reproducibility and Clinical Relevance of Quantitative Eeg Parameters in Cerebral Ischemia: A Basic Approach. *Clin Neurophysiol* (2009) 120(5):845-55. Epub 2009/04/21. doi: 10.1016/j.clinph.2009.02.171.
