# Supplementary material for: ELECTRA-STROKE: Electroencephalography controlled triage in the ambulance for acute ischemic stroke—Study protocol for a diagnostic trial
Source: Front Neurol. 2022 Oct 3;13:1018493. doi: 10.3389/fneur.2022.1018493 (PMC9576201; doi:10.3389/fneur.2022.1018493)
Supplement: Supplementary file 2 [file Data_Sheet_1.PDF]

ELECTRA-STROKE

EEG controlled triage in the ambulance for acute ischemic stroke

RESEARCH PROTOCOL

PROTOCOL TITLE: 'ELECTRA-STROKE: EEG controlled triage in the ambulance for acute ischemic stroke'

**Protocol ID**	NL65939.018.18
**Short title**	ELECTRA-STROKE
**EudraCT number**	Not applicable
**Version**	10.0
**Date**	20-04-2022
**Coordinating investigator**	Laura C.C. van Meenen, PhD-candidate<br>Amsterdam University Medical Centers<br>Location AMC<br>Department of Neurology, room H2-225<br>Meibergdreef 9, 1105 AZ Amsterdam<br>The Netherlands<br>Telephone: +31 20 5663447<br>Email: l.c.vanmeenen@amsterdamumc.nl
**Project leaders**	Dr. Jonathan Coutinho, neurologist<br>Amsterdam University Medical Centers<br>Location AMC<br>Department of Neurology, room H2-260<br>Meibergdreef 9, 1105 AZ Amsterdam<br>The Netherlands<br>Telephone: +31 20 566 2004<br>Email: j.coutinho@amsterdamumc.nl<br><br>Dr. Wouter Potters, technical physician<br>Amsterdam University Medical Centers<br>Location AMC<br>Dept. of Clin. Neurophysiology, room D2-127<br>Meibergdreef 9, 1105 AZ Amsterdam<br>The Netherlands<br>Telephone: +31 20 5663515<br>Email: w.v.potters@amsterdamumc.nl
**Principal investigator coordinating center (in Dutch: hoofdonderzoeker/ uitvoerder)**	Dr. Jonathan Coutinho, neurologist
**Study staff other participating centers**	VUMC<br>Marieke Visser, neurologist<br>Amsterdam UMC, location VUMC<br>Department of neurology

De Boelelaan 1117, 1081 HV Amsterdam
Telephone: 020-4442834
Email: mc.visser@vumc.nl

OLVG West
Renske van den Berg, neurologist
OLVG, location West
Department of Neurology
Jan Tooropstraat 164, 1061 AE Amsterdam
Telephone: 020-5108780
Email: r.vandenberg-vos@olvg.nl

Noordwest Ziekenhuisgroep, location Alkmaar
Patricia Halkes, neurologist
Noordwest Ziekenhuisgroep, location Alkmaar
Wilhelminalaan 12, 1815 JD Alkmaar
Telephone: 0725484444
Email: p.h.a.halkes@nwz.nl

Ambulance Amsterdam
Arjen Siegers, anesthesiologist and medical manager ambulance care Ambulance Amsterdam
Ambulance Amsterdam
Karperweg 19-25, 1075 LB Amsterdam
Telephone: 020-5709500
Email: asiegers@ambulanceamsterdam.nl

Witte Kruis Noord-Holland Noord
Martin Smeekes, director Safety Region Noord-Holland Noord, director Regional Ambulance Service Noord-Holland Noord
Hertog Aalbrechtweg 22, 1823 DL Alkmaar
Telephone: 072-5675010
Email: msmeekes@vrnhn.nl

Sponsor (in Dutch: verrichter/opdrachtgever)

Amsterdam University Medical Centers
Location AMC
Meibergdreef 9, 1105AZ Amsterdam
The Netherlands

Subsidising party

AMC Innovation Grant

Independent expert

Vincent Odekerken, neurologist
Amsterdam University Medical Centers
Location AMC

	Department of Neurology, room H2-228<br>Meibergdreef 9, 1105 AZ Amsterdam<br>The Netherlands<br>Telephone: +31 20 5663445<br>Email: v.j.odekerken@amc.nl
**Laboratory sites**	Not applicable
**Pharmacy**	Not applicable

PROTOCOL SIGNATURE SHEET

	Signature	Date
**Head of Department**		
Prof. dr. B.M.J. Uitdehaag, neurologist		
**Project leader**		
Dr. J. Coutinho, neurologist AMC		

TABLE OF CONTENTS

PROTOCOL TITLE ... 2
PROTOCOL SIGNATURE SHEET ... 5
TABLE OF CONTENTS ... 6
LIST OF ABBREVIATIONS AND RELEVANT DEFINITIONS ... 8
SUMMARY ... 9
1. INTRODUCTION AND RATIONALE ... 12
2. OBJECTIVES ... 14
3. STUDY DESIGN ... 14
4. STUDY POPULATION ... 16
4.1 Population (base) ... 16
4.2 Inclusion criteria ... 17
4.3 Exclusion criteria ... 18
4.4 Sample size calculation ... 18
5. TREATMENT OF SUBJECTS ... 20
6. INVESTIGATIONAL PRODUCT ... 21
7. NON-INVESTIGATIONAL PRODUCT ... 21
7.1 Name and description of non-investigational product ... 21
7.2 Summary of findings from non-clinical studies ... 21
7.3 Summary of findings from clinical studies ... 22
7.4 Summary of known and potential risks and benefits ... 22
8. METHODS ... 22
8.1 Study parameters/end points ... 22
8.1.1 Main study parameter/end point ... 22
8.1.2 Secondary study parameters/end points ... 22
8.2 Blinding ... 23
8.3 Study procedures ... 23

8.4 Withdrawal of individual subjects ... 24

8.5 Replacement of individual subjects after withdrawal ... 24

9. SAFETY REPORTING ... 24

9.1 Temporary halt for reasons of subject safety ... 24

9.2 AEs, SAEs and SUSARs ... 25

9.2.1 Adverse Events (AEs) ... 25

9.2.2 Serious Adverse Events (SAEs) ... 25

9.3 Follow up of adverse events ... 26

9.4 Safety committee ... 26

10. STATISTICAL ANALYSIS AND ALGORITHM DEVELOPMENT ... 26

11. ETHICAL CONSIDERATIONS ... 27

11.1 Regulation statement ... 27

11.2 Recruitment and consent ... 27

11.3 Incapacitated subjects ... 30

11.4 Benefits and risk assessment, group relatedness ... 31

11.5 Compensation for injury ... 31

11.6 Incentives ... 31

12. ADMINISTRATIVE ASPECTS AND MONITORING ... 31

12.1 Handling and storage of data and documents ... 31

12.2 Monitoring and Quality Assurance ... 31

12.3 Amendments ... 32

12.4 Annual progress report ... 32

12.5 Temporary halt and (premature) end of study report ... 32

13. RISK ANALYSIS ... 33

14. REFERENCES ... 33

LIST OF ABBREVIATIONS AND RELEVANT DEFINITIONS

AE	Adverse event
AIS	Acute ischemic stroke
AMC	Academic Medical Center, 1 of 2 Amsterdam UMC locations
Amsterdam UMC	Amsterdam University Medical Centers
AVG	General Data Protection Regulation (in Dutch: Algemene Verordening Gegevensbescherming)
CT	Computed Tomography
EEG	Electroencephalography
EMS	Emergency medical service
EVT	Endovascular thrombectomy
ER	Emergency room
METC	Medical research ethics committee (MREC); in Dutch: medisch ethische toetsingscommissie (METC)
MRI	Magnetic Resonance Imaging
mRS	Modified Rankin Scale
NIHSS	National Institute of Health Stroke Scale
OLVG West	Onze Lieve Vrouwe Gasthuis, locatie Amsterdam West
SAE	Serious adverse event
Sponsor	The sponsor is the party that commissions the organisation or performance of the research, for example a pharmaceutical company, academic hospital, scientific organisation or investigator. A party that provides funding for a study but does not commission it is not regarded as the sponsor, but referred to as a subsidising party.
VUMC	Vrije Universiteit Medisch Centrum, 1 of 2 Amsterdam UMC locations
WMO	Medical Research Involving Human Subjects Act (in Dutch: Wet Medisch-wetenschappelijk onderzoek met mensen)

SUMMARY

RATIONALE

Endovascular thrombectomy (EVT) is standard treatment for acute ischemic stroke (AIS) if there is a large vessel occlusion in the anterior circulation (LVO-a). Because of its complexity, EVT is performed in selected hospitals only. Currently, approximately half of EVT eligible patients are initially admitted to hospitals that do not provide this therapy. This delays initiation of treatment by approximately an hour, which decreases the chance of a good clinical outcome. Direct presentation of all patients with a suspected AIS in EVT capable hospitals is not feasible, since only approximately 7% of these patients are eligible for EVT. Therefore, an advanced triage method that reliably identifies patients with an LVO-a in the ambulance is necessary. Electroencephalography (EEG) may be suitable for this purpose, as preliminary studies suggest that slow EEG activity in the delta frequency range correlates with lesion location on cerebral imaging. Use of dry electrode EEG caps will enable relatively unexperienced paramedics to perform a reliable measurement without the EEG preparation time associated with 'wet' EEGs. Combined with algorithms for automated signal analysis, we expect the time of EEG recording and analysis to eventually be below five minutes, which would make stroke triage in the ambulance by EEG logistically feasible.

HYPOTHESIS

We hypothesize that dry electrode cap EEG can be used in patients with a suspected AIS to identify patients with an LVO-a in the ambulance.

OBJECTIVE

To determine the diagnostic accuracy of dry electrode cap EEG for diagnosis of LVO-a, when performed by paramedics in the ambulance in patients with a suspected AIS.

STUDY DESIGN

This diagnostic study consists of four phases:

Phase 1: Optimization of measurement time and software settings of the dry electrode cap EEG in a non-emergency setting in patients in whom a regular EEG is/will be performed for standard medical care.

Phase 2: Optimization of measurement time and software settings of the dry electrode cap EEG in patients close to our target population in a non-emergency setting.

Phase 3: Validation of several existing algorithms and development of one or more new algorithms for LVO-a detection, as well as optimization of logistics and software settings of the dry electrode EEG cap in patients close to our target population in an in-hospital emergency setting.

Phase 4: Validation of several existing algorithms and algorithms developed in phase 3 for LVO-a detection in patients with a suspected AIS in the ambulance, as well as assessment of technical and logistical feasibility of performing EEG with dry electrode caps in patients with a suspected AIS in the ambulance.

STUDY POPULATION

Phase 1: Patients in the outpatient clinic of the Clinical Neurophysiology department of the AMC, in whom a regular EEG has been/will be performed for standard medical care.

Phase 2: Patients with an AIS admitted to the Neurology ward of the coordinating hospital with an LVO-a (after reperfusion therapy).

Phase 3: Patients with a suspected AIS in the emergency room (ER) of the coordinating hospital (before reperfusion therapy).

Phase 4: Patients with a suspected AIS in the ambulance.

INTERVENTION

Performing a dry electrode cap EEG (in phase 1 in the outpatient clinic, in phase 2 during hospital admission, in phase 3 in the ER and in phase 4 in the ambulance).

MAIN END POINTS

- Primary end point: the area under the receiver operating characteristics (ROC) curve of the theta/alpha ratio for diagnosis of LVO-a, based on dry electrode EEG performed by paramedics in the ambulance in suspected AIS patients;
- Secondary end points:

- Sensitivity, specificity, positive predictive value (PPV) and negative predictive value (NPV) of the theta/alpha ratio, and test characteristics of other existing EEG data based algorithms for LVO-a detection (e.g. Weighted Phase Lag Index, delta/alpha ratio);
- Logistical and technical feasibility of paramedics performing dry electrode cap EEG in the ambulance in suspected AIS patients ;
- Developing one or more novel EEG data based algorithms with optimal diagnostic accuracy for LVO-a detection in suspected AIS patients with ambulant dry electrode cap EEG.

NATURE AND EXTENT OF THE BURDEN AND RISKS ASSOCIATED WITH PARTICIPATION, BENEFIT AND GROUP RELATEDNESS

A single EEG is performed on each patient. The EEG is a safe, non-invasive and painless procedure that is used regularly in standard medical practice. It does not involve electromagnetic radiation. The use of dry electrodes makes the procedure less time-consuming. After optimization of measurement time in phase 1 and 2, the procedure will take less than five minutes in total. Therefore, when the measurements are performed in an emergency setting in phases 3 and 4, the procedure can be completed within the time it takes the treating physician or paramedic to perform their regular work-up. This way, initiation of treatment will not be delayed. We expect the dry electrode cap to cause no to minimal discomfort, and only during the measurement. We will use CE marked products for performing the dry electrode cap EEGs. The results of the EEG will only be analyzed after admission or discharge, and will therefore not influence the choice of hospital by the paramedic. As soon as feasible, preferably within 72 hours after arrival at the hospital or at discharge (whichever comes first), deferred informed consent will be asked. If informed consent is given, a CRF will be filled out containing information on patient characteristics, medical history, medication use, physical and neurological examination performed by the treating physician, results of imaging studies, diagnosis and treatment as well as logistical and technical information, obtained from the patient, the treating physician and the ambulance service. There are no follow up visits. For the patient, there is no benefit of participation in the study.

1. INTRODUCTION AND RATIONALE

Acute ischemic stroke (AIS) is a major cause of mortality and morbidity worldwide, affecting approximately 1 per 1000 people per year[1]. In the Netherlands, 20.000 people are hospitalized with AIS each year, of whom 25-30% die because of the stroke[2,3]. Since the 1990's, intravenous thrombolysis (IVT) has been standard treatment for AIS[4]. In 2015, there was a breakthrough in treatment of AIS with the publication of 5 large randomized controlled trials showing the effectiveness of endovascular thrombectomy (EVT) in patients with a large vessel occlusion in the anterior circulation (LVO-a)[5]. Since then, EVT has been become standard therapy for this population until 6 hours after stroke onset, and as of recently, for a selected population, up to 24 hours[5-7]. It is important to initiate treatment as soon as possible, as this increases the chance of a good clinical outcome[8].

Because of its complexity, EVT is performed only in selected hospitals. Currently, approximately half of EVT eligible patients are initially admitted to a hospital that does not provide this therapy. After the initial work-up, these patients are transferred to an EVT capable hospital. This delays initiation of treatment by approximately an hour, which decreases the absolute chance of functional independence 3 months after the stroke by 5-8%[8,9]. An advanced triage method that reliably identifies patients with an LVO-a in the ambulance is necessary, so that these patients can be directly transported to an EVT capable stroke center.

A triage method for this purpose needs to have both high specificity and high sensitivity for LVO-a. The sensitivity of the diagnostic method is important, because a false negative test result means that the patient would be transported to a non EVT capable hospital, delaying his/her treatment. However, we deem the specificity of the method even more important, for the following reasons. Currently, EVT capable hospitals are overburdened because many patients with a suspected stroke are directly brought to these hospitals, as paramedics often do not want to take the chance of bringing a patient to the 'wrong' hospital. However, only a small part of these patients, circa 7%, is eligible for EVT. This causes EVT capable hospitals to have low capacity for admission of patients that do need specialized care. Additionally, patients with expected stroke currently are first evaluated in the emergency room where they undergo diagnostic procedures, and are then brought to the angiography room to undergo EVT. This takes approximately an hour from entering the hospital to the start of treatment (not all this time is due to diagnostic procedures, but also to other logistical issues that are not addressed in this study). Currently, the angiography room is not being prepared for every patient that is presented to the

emergency room with a suspected stroke, because of the low a priori chance of eligibility for EVT in patients with a suspected stoke. A diagnostic method with high specificity will make it possible, in patients in whom a high likelihood of an LVO-a has been determined in the ambulance, to minimize (or maybe even leave out) diagnostic procedures in the emergency room and to present these patients directly to the angiography room, in order to save time. Several methods for determining the likelihood of an AIS with an LVO-a have been proposed. However, none of these have been found to be suitable for prehospital triage. Clinical scales, containing items for scoring the severity of neurological deficit, are difficult to be reliably applied by paramedics and have low diagnostic accuracy[10]. Another option for pre-hospital stroke triage that has been explored, is equipping ambulances with an imaging system for performing non-contrast CT and CT angiography to diagnose LVO-a and, in some studies, give IVT. However, its efficacy has not been proven and there are concerns regarding the safety and cost-effectiveness of this method[11]. An effective and safe prehospital triage method for stroke that can be reliably performed and analyzed by paramedics has not yet been found.

Electroencephalography (EEG) may be suitable for prehospital triage, as preliminary studies suggest that slow EEG activity in the delta frequency range correlates with lesion location on cerebral imaging[12,13]. Another study has suggested using (a)symmetry in brain activity for monitoring of stroke patients[14]. EEG is already being used in clinical practice for monitoring for signs of cerebral ischemia during carotid surgery while the carotid artery is clamped, and can predict the likelihood of postoperative neurological deficit with high specificity (84-92%)[15,16]. This suggests that the occlusion of a large artery, similar to clamping of the carotid artery in its effect on cerebral blood supply, could also be predicted by EEG. One case-control study found that in patients with a recent AIS, the Brain Symmetry Index – an index that expresses EEG asymmetry (the higher the index, the more asymmetrical is the EEG signal) – positively correlated with severity of neurological deficits[17]. Other case-control studies have shown that ratios between slow and fast EEG activity, such as the delta/alpha ratio, can be used to discriminate between AIS patients and healthy controls with high sensitivity and specificity[18,19]. The Weighted Phase Lag Index, a measure for functional connectivity between different areas of the brain, has also been proposed for this purpose[19]. Although a regular EEG measurement takes approximately an hour, mostly due to preparation time, using dry electrode EEG caps decreases preparation time dramatically[20]. Combined with algorithms for automated signal analysis, we expect the time of a single EEG recording and analysis to be below 5 minutes, which will make stroke triage in the ambulance by EEG logistically feasible. Automated analysis would make it unnecessary for paramedics to be schooled in EEG interpretation;

combined with the easily applicable dry electrode cap, this should enable relatively unexperienced paramedics to perform a reliable measurement.

To our knowledge, no study of the diagnostic accuracy of EEG for LVO-a has been performed. We hypothesize that EEG can accurately predict the likelihood of the presence or absence of an LVO-a in patients with a suspected AIS when performed in the ambulance by paramedics. If this is indeed true, then EEG could be used for triage for patients with a suspected AIS, in a similar way the electrocardiography (ECG) is currently used for triage in patients with suspected myocardial infarction in the ambulance: by bringing patients with a high likelihood of necessity of endovascular therapy directly to an EVT capable center.

2. OBJECTIVES

The primary objective of this diagnostic study is to determine the diagnostic accuracy of dry electrode cap EEG for diagnosis of LVO-a, when performed by paramedics in the ambulance in patients with a suspected AIS.

The secondary objectives of this study are:

- Determining the diagnostic accuracy for LVO-a detection of several existing EEG data based algorithms (e.g. Weighted Phase Lag Index, delta/alpha ratio);

- Assessing the logistical and technical feasibility of paramedics performing dry electrode cap EEG in the ambulance in suspected AIS patients;

- Developing one or more novel EEG data based algorithms with an optimal diagnostic accuracy for LVO-a detection in suspected AIS patients with ambulant dry electrode cap EEG.

3. STUDY DESIGN

The ELECTRA-STROKE is an investigator-initiated, diagnostic study, that consists of four phases (see below).

STUDY PHASE 1: OPTIMIZING MEASUREMENT TIME, SOFTWARE SETTINGS AND DATA TRANSFER LOGISTICS IN PATIENTS IN WHOM A REGULAR EEG HAS BEEN/WILL BE PERFORMED FOR STANDARD MEDICAL CARE IN A NON-EMERGENCY SETTING

This phase will be carried out in the outpatient clinic of the department of Clinical Neurophysiology of a single center (Amsterdam UMC, location AMC). We will perform dry electrode cap EEGs on patients in whom a regular EEG has been/will be performed for standard medical care. We will compare the regular EEG to the dry electrode cap EEG and this way try to optimize the dry electrode cap EEG measurement, software settings, data transfer logistics and measurement time in a population of patients less vulnerable than our target population, in a non-emergency setting.

STUDY PHASE 2: OPTIMIZING MEASUREMENT TIME, SOFTWARE SETTINGS AND DATA TRANSFER LOGISTICS IN PATIENTS CLOSE TO TARGET POPULATION IN A NON-EMERGENCY SETTING.

This phase will be carried out in the brain care unit of a single center (Amsterdam UMC, location AMC). We will perform dry electrode cap EEGs in a population close to our target population – patients that have been admitted with an AIS and an LVO-a – in a non-emergency setting. We will test technical feasibility of the dry electrode cap EEG in patients that are, in part, unable to cooperate with the procedure (e.g. patients that because of lowered consciousness or agitation will not keep their head still).

STUDY PHASE 3: VALIDATION OF EXISTING ALGORITHMS AND DEVELOPMENT OF NEW ALGORITHM(S) IN PATIENTS IN THE EMERGENCY ROOM

This phase will be carried out in the ER of a single center (Amsterdam University Medical Centers, location AMC). In this phase, we will perform dry electrode cap EEGs in patients that are presented to the ER of the coordinating hospital with a clinically suspected AIS, according to the paramedic that presents the patient, or with a known LVO-a. The last category of patients have undergone an initial work-up in a primary stroke center, where the diagnosis LVO-a has been made, and are transferred to our hospital to undergo EVT. The EEG data of this ‘enriched’ population, with a higher incidence of LVO-a compared to the primary target population, will be used to validate several existing algorithms and to develop one or more new algorithms for diagnosis of LVO-a.

STUDY PHASE 4: VALIDATION OF ALGORITHMS IN THE AMBULANCE

This phase is the only multi-center phase of this study. It will be carried out in the ambulances of one ambulance station in Amsterdam and one ambulance station in Alkmaar. These ambulances bring patients with a suspected acute stroke to the following hospitals: AMC, VUMC and OLVG West and Noordwest Ziekenhuisgroep, location Alkmaar. These will therefore be the centers participating in this study phase, as well as the Ambulance Amsterdam. The EEG data will be used to validate several existing algorithms (among which is the Brain Symmetry Index) and the algorithm(s) developed in phase 3. Technical and logistical feasibility of performing ambulant EEGs in patients with a suspected AIS in the ambulance will also be assessed.

Phase 4 will start with a ‘run in’ phase. The objective of this phase is to assess technical and logistical feasibility of performing ambulant EEGs in patients with a suspected AIS, before commencing inclusion of the prespecified study population. Inclusion will not be consecutive at this stage.

The four phases will not necessarily run consecutively; they may also, in part, run simultaneously. Each phase consists of performing a single dry electrode cap EEG per patient and collecting information on patient characteristics, medical history, medication use, physical and neurological examination performed by the treating physician, imaging studies, diagnosis and treatment as well as logistical and technical information, obtained from the patient, the Emergency Medical Service (EMS) and the treating physician. There are no follow up visits. The study will run until the number of patients required according to the sample size calculation have been included for each phase.

4. STUDY POPULATION

4.1 POPULATION (BASE)

STUDY PHASE 1

We will recruit patients in whom a regular EEG has been/will be performed for standard medical care in the outpatient clinic of the Clinical Neurophysiology department of the AMC, who meet the criteria described in chapter 4.2 and 4.3.

STUDY PHASE 2

We will recruit patients with a that have been admitted to the hospital with an LVO-a, who meet the criteria described in chapter 4.2 and 4.3.

STUDY PHASE 3

We will recruit patients with a suspected AIS or a known LVO-a in the ER, who meet the criteria described in chapter 4.2 and 4.3.

STUDY PHASE 4

We will recruit patients with a suspected AIS in the ambulance, who meet the criteria described in chapter 4.2 and 4.3.

4.2 INCLUSION CRITERIA

STUDY PHASE 1

- Age of 18 years or older;

- Patient is in the outpatient clinic of the Clinical Neurophysiology department of the AMC, because a regular EEG has been/will be performed on him/her for standard medical care;

- Written informed consent by patient.

STUDY PHASE 2

- A diagnosis of acute ischemic stroke caused by a large vessel occlusion in the anterior circulation (intracranial carotid artery or proximal (M1/M2) middle cerebral artery confirmed by neuro-imaging (CTA or MRA);

- Stroke onset <72 hours before expected time of performing EEG;

- Age of 18 years or older;

- Written informed consent by patient or legal representative.

STUDY PHASE 3

- Suspected acute ischemic stroke, as judged by the paramedic presenting the patient to the ER <u>or</u> known AIS with an LVO-a;

- Onset of symptoms or, if onset was not witnessed, last seen well <24 hours ago;

- Age of 18 years or older;

- Written informed consent by patient or legal representative (deferred).

STUDY PHASE 4

- Suspected acute ischemic stroke as judged by the attending paramedic;

- Onset of symptoms or, if onset not witnessed, last seen well <24 hours ago;

- Age of 18 years or older;

- Written informed consent by patient or legal representative (deferred).

4.3 EXCLUSION CRITERIA

STUDY PHASE 1

- Injury or active infection of electrode cap placement area.

STUDY PHASE 2

- Injury or active infection of electrode cap placement area.

STUDY PHASE 3

- Injury or active infection of electrode cap placement area.

STUDY PHASE 4

- Injury or active infection of electrode cap placement area.

4.4 SAMPLE SIZE CALCULATION

STUDY PHASE 1

For study phase 1, where we aim to optimize measurement time, software settings and data transfer logistics in patients in the outpatient clinic of the Clinical Neurophysiology department of the AMC, we

plan to include 20 patients or less, depending on the judgement of the investigators of when the optimal settings and logistics have been found.

STUDY PHASE 2

For study phase 2, where we aim to optimize measurement time, software settings and data transfer logistics in a population of patients with an AIS in a non-emergency setting, we plan to include 20 patients or less, depending on the judgement of the investigators of when the optimal settings and logistics have been found.

STUDY PHASE 3

For study phase 3, where we aim to validate existing algorithms and to develop one or more new algorithms, we plan to include 60 patients with an LVO-a. Because the coordinating center, where this phase will be conducted, is the only hospital in its region performing EVT, many patients with an LVO-a are transported here. In the population of suspected ischemic stroke patients in this hospital's ER, including patients transferred for EVT, the incidence of LVO-a is approximately 20% based on data from our own stroke database. Therefore, we aim to include 300 patients in this phase (approximately 60 patients with an LVO-a and 240 patients without an LVO-a) or less, depending on the judgement of the investigators of when the goal of this study phase is achieved.

STUDY PHASE 4

For study phase 4, where we aim to validate existing algorithms and one or more new algorithms for LVO-a detection, our primary outcome measure is the specificity of the dry electrode cap EEG in a population of patients with a suspected AIS. Sample size calculation is based on an expected specificity of 70% for LVO-a. Originally, sample size calculation was based on an expected LVO-a rate of 7% and a dropout rate of 20%. However, an interim analysis, based on 137 suspected stroke patients in whom an EEG was performed in the ambulance, showed an actual incidence of LVO-a of 5% and a dropout rate of 58%. Patients considered as a 'drop out' had insufficient EEG data quality (n=72), onset of symptoms >24 hours before EEG registration (n=4) or did not give consent for participation in the study (n=3). The EEG data quality increased over the course of the study, so we expect a lower dropout rate for future EEG registrations. Based on these interim data, we adjusted the sample size for phase 4 of the study as follows.

With an incidence of LVO-a of 5% among patients with a clinically suspected AIS in the ambulance, an alpha of 0.05, and a maximum margin of error of 7%, 174 patients with a suspected AIS are required[21]. Assuming a dropout rate of 55%, we intend to recruit 386 patients with a suspected AIS. A patient is considered a ‘drop out’ if no informed consent is/can be obtained, if no information on the diagnosis made by the treating physician is/can be obtained, or if the EEG measurement is of insufficient technical quality, as judged by the clinical neurophysiologist. Patients included in the ‘run in’ phase are excluded from the primary analysis and thus not included in our sample size calculation. In the ‘run in’ phase, we will additionally include 50 patients or less.

5. TREATMENT OF SUBJECTS

STUDY PHASE 1

After informed consent is obtained, a single dry electrode cap EEG (see chapter 6) will be performed in each patient. We will compare the regular EEG to the dry electrode cap EEG and collect information on patient characteristics, medical history and medication use. There are no follow up visits.

STUDY PHASE 2

After informed consent is obtained, a single dry electrode cap EEG (see chapter 6) will be performed in each patient. Additionally we will collect information on patient characteristics, medical history, medication use, physical and neurological examination performed by the treating physician, imaging studies, diagnosis and treatment, obtained from the patient and the treating physician. There are no follow up visits.

STUDY PHASE 3

Directly at presentation in the ER, a single dry electrode cap EEG (see chapter 6) will be performed in each patient. As soon as feasible, preferably within 72 hours after arrival at the hospital or at discharge (whichever comes first), informed consent is asked (deferred consent, see chapter 10). If informed consent is obtained, we will collect information on patient characteristics, medical history, medication use, physical and neurological examination performed by the treating physician, imaging studies, diagnosis and treatment as well as logistical and technical information, obtained from the patient, the EMS and the treating physician. There are no follow up visits.

STUDY PHASE 4

In the ambulance, a single dry electrode cap EEG (see chapter 6) will be performed in each patient. As soon as feasible, preferably within 72 hours after arrival at the hospital or at discharge (whichever comes first), informed consent is asked (deferred consent, see chapter 10). If informed consent is obtained, we will collect information on patient characteristics, medical history, medication use, physical and neurological examination performed by the treating physician, imaging studies, diagnosis and treatment as well as logistical and technical information, obtained from the patient, the EMS and the treating physician. There are no follow up visits.

6. INVESTIGATIONAL PRODUCT

This is not applicable, since no investigational product(s) will be used in this study.

7.NON-INVESTIGATIONAL PRODUCT

7.1 NAME AND DESCRIPTION OF NON-INVESTIGATIONAL PRODUCT

We plan to use the Waveguard™ dry electrode EEG cap and compatible eego™ amplifiers, developed by ANT Neuro B.V. Netherlands and all CE marked as medical devices in the European Union (see appendices 1 and 2). The dry electrode cap records the EEG signal; the amplifier is used to amplify the EEG signal and reduce artefacts. All products will be used within the intended use as described in the user manuals (see appendices 5, 6, 7, 8 and 9). We will use CE marked NeuroCenter® EEG software (CE class IIa) from Clinical Science Systems (ISO 9001 and ISO 13485 certified) and CE marked eego™ software (CE class IIa) from ANT Neuro B.V. to acquire the signals from the eego™ compatible amplifiers (see appendix 3 and 4).

In a subgroup of patients, who will be included in study phase 3, we plan to use Ambu® Neuroline 720 electrodes together with the Refa multichannel amplifier by TMSi (Twente Medical Systems International B.V. Netherlands), which are CE marked as medical devices in the European Union (see appendices 10 and 11). The electrodes record the EEG signal; the amplifier is used to amplify the EEG signal and reduce artefacts. All products will be used in accordance with the intended use as described in the user manuals (see appendices 12 and 13).

7.2 SUMMARY OF FINDINGS FROM NON-CLINICAL STUDIES

This is not relevant for the Waveguard™ dry electrode cap, eego™ amplifier, Neuroline 720 electrodes and Refa amplifier as they are all CE marked products that have been safely used in clinical studies (see chapter 7.3). NeuroCenter® EEG software and the eego™ software are both a CE class IIa product.

7.3 SUMMARY OF FINDINGS FROM CLINICAL STUDIES

The Waveguard™ dry electrode cap, eego™ amplifier, Neuroline 720 electrodes and Refa amplifier have all been safely used in several clinical studies, with no reported adverse events[22-31]. A small clinical study has shown that the Waveguard's signal quality is similar to that of the conventional 128-channel wet (gel/paste) EEG[22]. However, to our knowledge, no study of the diagnostic accuracy of dry electrode cap EEG (or EEG in general) for LVO-a has been performed.

7.4 SUMMARY OF KNOWN AND POTENTIAL RISKS AND BENEFITS

The benefit of using either a dry electrode EEG cap or Neuroline 720 electrodes is that they are easily applicable and drastically reduce preparation time, which makes them suitable for use in an emergency setting. Since EEG is a safe, non-invasive and painless procedure that is used regularly in standard medical practice and we will use CE marked products for our measurements, we expect no health risks and no to minimal discomfort during a short period of time associated with participation[32]. The dry electrode EEG caps will be cleaned and disinfected as recommended in the user manual (appendix 5) and the disposable Neuroline 720 electrodes will be for single use only.

8. METHODS

8.1 STUDY PARAMETERS/END POINTS

8.1.1 MAIN STUDY PARAMETER/END POINT

The primary end point of the study is the area under the ROC curve of the theta/alpha ratio for diagnosis of LVO-a, based on dry electrode EEG performed by paramedics in the ambulance in suspected AIS patients.

8.1.2 SECONDARY STUDY PARAMETERS/END POINTS

Secondary end points of this study are:

- Sensitivity, specificity, PPV and NPV of the theta/alpha ratio, and test characteristics of other existing EEG data based algorithms for LVO-a detection (e.g. Weighted Phase Lag Index, delta/alpha ratio);

- Logistical and technical feasibility of paramedics performing dry electrode cap EEG in the ambulance in suspected AIS patients;

- Developing one or more novel EEG data based algorithms with an optimal diagnostic accuracy for LVO-a detection in suspected AIS patients with ambulant dry electrode cap EEG.

8.2 BLINDING

In this diagnostic accuracy study, the treating clinical team (in all phases) and the paramedics (in phase 4) are blinded for the outcome of the EEG.

8.3 STUDY PROCEDURES

All collected data and study procedures are listed per study phase in the following table. There are no follow up visits. Items that are **not** part of standard medical procedure/treatment are bold.

	Phase 1	Phase 2	Phase 3	Phase 4
Patient characteristics*	**At baseline**	At baseline	<24 hours after arrival in hospital or at discharge (whichever comes first)	<24 hours after arrival in hospital or at discharge (whichever comes first)
Past medical history	**At baseline**	At baseline	<24 hours after arrival in hospital or at discharge (whichever comes first)	<24 hours after arrival in hospital or at discharge (whichever comes first)
Medication use	**At baseline**	At baseline	<24 hours after arrival in hospital or at discharge (whichever comes first)	<24 hours after arrival in hospital or at discharge (whichever comes first)
Pre-stroke modified Rankin Scale score	NA	**At baseline**	**<24 hours after arrival in hospital or at discharge (whichever comes first)**	**<24 hours after arrival in hospital or at discharge (whichever comes first)**
Physical and neurological examination by treating physician	NA	At arrival in ER, data collected retrospectively at baseline	At arrival in ER	At arrival in ER
National Institute of	NA	**At arrival in ER, data**	**At arrival in ER**	**At arrival in ER**

Health Stroke Scale (NIHSS) score		**collected retrospectively at baseline**		
Brain imaging results, if available	NA	At arrival in ER, data collected retrospectively at baseline	At arrival in ER (if necessary, as judged by treating physician)	At arrival in ER (if necessary, as judged by treating physician)
Dry electrode cap EEG	**At baseline**	**At baseline**	**At arrival in ER**	**At arrival of ambulance**
Final diagnosis, as judged by treating physician	NA	At baseline	At discharge	At discharge
Treatment with IVT and/or EVT	NA	Data collected retrospectively at baseline	At arrival in ER (if necessary, as judged by treating physician)	At arrival in ER (if necessary, as judged by treating physician)
Times regarding stroke logistics**	NA	**Data collected retrospectively at baseline**	**Collected during first 24 hours after symptom onset**	**Collected during first 24 hours after symptom onset**

*Age, sex, right/left-handedness, estimated quantity and length of hair on the head of the participant.
**Time of: last seen well or witnessed stroke onset, 112-call, ambulance arrival on site, ambulance departure to hospital, ambulance arrival at hospital, start neuro-imaging (if any), start IVT/EVT (if any), transfer times from other primary hospital (if applicable).

8.4 WITHDRAWAL OF INDIVIDUAL SUBJECTS

Subjects can leave the study at any time for any reason if they wish to do so without any consequences. The investigator can decide to withdraw a subject from the study for urgent medical reasons.

8.5 REPLACEMENT OF INDIVIDUAL SUBJECTS AFTER WITHDRAWAL

Since our sample size calculation includes an expected dropout rate, we will not replace subjects that have withdrawn from the study. Record will be kept of all reasons for withdrawal and these will be reported along with the study results.

9. SAFETY REPORTING

9.1 TEMPORARY HALT FOR REASONS OF SUBJECT SAFETY

In accordance to section 10, subsection 4, of the WMO, the sponsor will suspend the study if there is sufficient ground that continuation of the study will jeopardise subject health or safety. The sponsor will notify the accredited METC without undue delay of a temporary halt including the reason for such an action. The study will be suspended pending a further positive decision by the accredited METC. The investigator will take care that all subjects are kept informed.

9.2 AES, SAES AND SUSARS

9.2.1 ADVERSE EVENTS (AES)

Adverse events are defined as any undesirable experience occurring to a subject during the study, whether or not considered related to the investigational product or the intervention. All adverse events reported spontaneously by the subject or observed by the investigator or his/her staff will be recorded.

9.2.2 SERIOUSADVERSE EVENTS (SAES)

A serious adverse event is any untoward medical occurrence or effect that:

- results in death;
- is life threatening (at the time of the event);
- requires hospitalisation or prolongation of existing inpatients' hospitalisation;
- results in persistent or significant disability or incapacity;
- any other important medical event that did not result in any of the outcomes listed above due to but could have been based upon appropriate judgement by the investigator.

The sponsor will report the SAEs through the web portal *ToetsingOnline* to the accredited METC that approved the protocol, within 15 days of first knowledge of the SAE.

Stroke often results in complications such as infections, delirium, constipation, decubitus wounds, swallowing difficulties and falls, as well as in hospitalization and death. Because we expect no health risk related to participation in this study, these events will be reported to the accredited METC that approved the protocol in a yearly line listing until the last patient has completed the study.

Furthermore, the following situations will not be reported as (S)AEs:

- Elective hospitalization for pre-existing conditions that have not been exacerbated by study treatment as judged by the clinical investigator and where admission did not take longer than anticipated;
- Admission for diagnosis or therapy of a condition that existed before inclusion to this study and has not increased in severity or frequency as judged by the clinical investigator;
- Anticipated day-to-day fluctuations of pre-existing disease(s) or condition(s) present at the start of the study that do not worsen.

9.3 FOLLOW UP OF ADVERSE EVENTS

All AEs will be followed until they have abated, or until a stable situation has been reached. Depending on the event, follow up may require additional tests or medical procedures as indicated, and/or referral to the general physician or a medical specialist. SAEs will be reported if they occur within 1 hour after completion of the EEG-measurement.

9.4 SAFETY COMMITTEE

Installation of a safety committee is not deemed necessary, considering that EEG is a safe, non-invasive and painless procedure that is used regularly in standard medical practice and we are using CE marked products only.

10. STATISTICAL ANALYSIS AND ALGORITHM DEVELOPMENT

STUDY PHASE 1 AND 2

For phase 1 and 2 of this study, the obtained data will not be statistically analyzed, for these phases are meant to gain technical and logistical information on the products being used in a non-emergency setting.

STUDY PHASE 3

For phase 3 of this study, we will determine the diagnostic accuracy of several existing and one or more self-developed EEG data based algorithms for LVO-a detection by comparing the proportions of patients with and without an LVO-a as predicted by EEG to the proportion of patients with and without an LVO-a as diagnosed by the treating physician. A receiver operating characteristic (ROC) curve will be plotted for each algorithm and the optimal cut-off value for each algorithm will be determined. For these cut off values, the sensitivity, specificity, negative predictive value and positive predictive value will be reported with 95% confidence intervals.

ALGORITHM DEVELOPMENT

Data of phase 3 and phase 4 will be used to develop one or more new EEG based algorithms for LVO-detection. We will create artificial intelligence (AI) based algorithm(s). First, we will create a logistic

regression model based on standard EEG features, e.g. time domain characteristics, power spectral density in different frequency bands, coherence measures, and entropy measures. This will be the reference to assess the improvement of the novel AI based models. Second, we will work on EEG classification algorithms based on random forests and support vector machines, using the same EEG features as input. Finally, to support future studies, we will explore new methods that use raw EEG data as input, such as convolutional or recurrent neural networks[33-35].

STUDY PHASE 4

For phase 4 of this study, we will determine the diagnostic accuracy of several existing and one or more self-developed EEG data based algorithms for LVO-a detection by comparing the proportions of patients with and without an LVO-a as predicted by EEG to the proportion of patients with and without an LVO-a as diagnosed by the treating physician. An ROC curve will be plotted for each algorithm and the optimal cut-off value will be determined. For this cut off value, the sensitivity, specificity, negative predictive value and positive predictive value will be reported with 95% confidence intervals.

11. ETHICAL CONSIDERATIONS

11.1 REGULATION STATEMENT

This study will be conducted according to the principles of the Declaration of Helsinki (October 2013) and in accordance with the Medical Research Involving Human Subjects Act (WMO).

11.2 RECRUITMENT AND CONSENT

STUDY PHASE 1

For phase 1 of this study, patients will be recruited by the investigators and physicians of the departments of Neurology and Clinical Neurophysiology of the coordinating hospital. Patients fulfilling the in- and exclusion criteria will be given the study information letter and, after having been giving time to read, think about and ask questions regarding the content of the letter, will be asked for written informed consent. After informed consent is obtained, the patient will be included in the study.

STUDY PHASE 2

For phase 2 of this study, patients will be screened by the investigators and physicians of the department of Neurology. Patients fulfilling the in- and exclusion criteria of this study, or their legal representative (see chapter 10.3), will be given the study information letter and, after having been giving time to read, think about and ask questions regarding the content of the letter, will be asked for written informed consent. After informed consent is obtained, the patient will be included in the study.

STUDY PHASE 3

For phase 3 of this study, patients will be screened for eligibility in the ER by the investigators. In patients fulfilling the in- and exclusion criteria, an EEG will be performed before obtaining informed consent, and the diagnosis made by the treating physician will be registered. We will withhold any further study procedures until informed consent is obtained. Afterwards, as soon as feasible and preferably within 72 hours after arrival at the hospital or at discharge (whichever comes first), the patient or legal representative (see chapter 10.3) is informed and asked for informed consent by the investigator. If informed consent is granted, we will use the data that have already been obtained and continue the study procedures. If informed consent is not granted, we will ask the patient or legal representative for consent to use the data that have been collected up to that point for this study. If this is granted, we will use the data that have already been obtained but will not proceed with further study procedures. If this is denied, the obtained data will be destroyed. Figure 1 shows the procedure of obtaining deferred consent. If the patient is deceased before informed consent can be asked from the patient or the legal representative, the data that have been collected will be used for the study without having obtained informed consent.

STUDY PHASE 4

For phase 4 of this study, patients will be screened for eligibility in the ambulance by the first responding paramedic. In patients fulfilling the in- and exclusion criteria, an ambulant EEG will be performed before obtaining informed consent, and the diagnosis made by the treating physician will be registered. We will withhold any further study procedures until informed consent is obtained. As soon as feasible and preferably within 72 hours after arrival at the hospital or at discharge (whichever comes first), the patient or legal representative (see chapter 10.3) is informed and asked for informed consent by the investigator. If informed consent is granted, we will use the data that have already been obtained and continue the study procedures. If informed consent is not granted, we will ask the patient or legal

representative for consent to use the data that have been collected up to that point for this study. If this is granted, we will use the data that have already been obtained but do not proceed with further study procedures. If this is denied, the obtained data will be destroyed. Figure 1 shows the procedure of obtaining deferred consent. If the patient is deceased before informed consent can be asked from the patient or the legal representative, the data that have been collected will be used for the study without having obtained informed consent.

ETHICAL CONSIDERATIONS REGARDING DEFERRED CONSENT

Informed consent is fundamental for patient participation in any type of research. However, in acute stroke research, the 'time is brain' principle conflicts with this. For study procedures that need to be performed in the acute setting, asking informed consent in a way that the patient or legal representative has the time to calmly consider the information and ask questions means losing valuable time: an hour delay in initiation of reperfusion therapy (IVT or EVT) in AIS causes a 5-8% decrease in chance of functional independence 3 months after the stroke[8,9]. Also, asking for informed consent in the acute setting is unlikely to result in a decision that is well thought through, because the patient or legal representative may be overwhelmed and under psychological stress[36]. Finally, many patients with an AIS are unable to comprehend information regarding study participation due to lowered consciousness, aphasia or anosognosia (not realizing the severity of one's own disease, often due to a lesion of the non-dominant cerebral hemisphere). Only including patients that are capable of giving informed consent would mean selecting a sub group of patients with relatively mild neurological symptoms that is not representative of the population of patients with an LVO-a. A legal representative is often not present in the acute phase[37]. For the above mentioned reasons, several acute stroke trials have been conducted where informed consent was not required prior to randomization[38]. According to the Medical Research Human Subjects Act (in Dutch: WMO), for research that can only be conducted in an emergency setting it is allowed to perform study procedures without prior informed consent, if inclusion in the study may benefit the person in urgent need of medical treatment, for as long as the circumstances preventing the giving of consent exist.

Performing an EEG in patients with a suspected AIS in the short time window prior to initiation of treatment is necessary in this study, because the objective is to study the diagnostic accuracy of the ambulant EEG for LVO-a. As soon as the occlusion is being treated (by IVT and/or IAT), this is no longer possible. Although participation in this study does not benefit the patient, it is very important that a

reliable stroke triage method is found for stroke patients in the long term, as described in chapter 1. Considering that EEG is a safe, non-invasive and painless procedure that is used regularly in standard medical practice and does not involve electromagnetic radiation, alongside the above mentioned reasons, we deem it ethical to perform the EEG and register the diagnosis prior to asking informed consent, while withholding all other study procedures until informed consent is obtained. We will ask informed consent as soon as feasible, preferably within 72 hours after arrival at the hospital or at discharge (whichever comes first). If informed consent is not granted, we will ask the patient or legal representative for consent to use the earlier obtained data for this study. If this is granted, we will use the data that have already been obtained but will not proceed with further study procedures. If this is denied, the obtained data will be destroyed. Figure 1 shows the procedure of obtaining deferred consent.

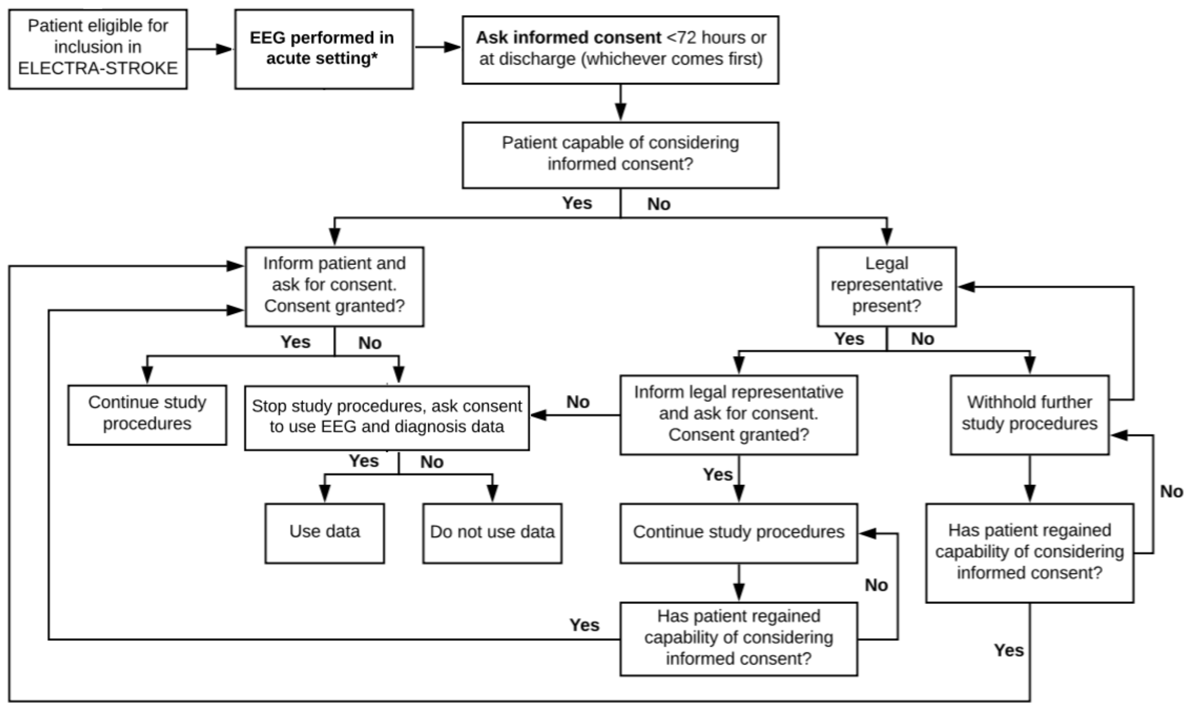

Figure 1: Flow chart showing process of obtaining deferred consent for phase 3 and 4 of the study. Acute setting is in the ER for phase 3 and in the ambulance for phase 4.

11.3 INCAPACITATED SUBJECTS

Due to lowered consciousness, aphasia or anosognosia (not realizing the severity of one's own disease, often due to a lesion of the non-dominant cerebral hemisphere), patients with an AIS frequently are

unable to comprehend information regarding study participation. Therefore, they are often not able to give or to deny informed consent. It is essential that these patients are included in the study, as excluding them would mean selecting a sub group of patients with relatively few neurological symptoms that is not representative of the population of patients with an LVO-a. If a patient is incapacitated, their legal representative (according to the WMO) will be asked for informed consent. If an incapacitated patient becomes capable of considering informed consent during admission, informed consent will be asked. If denied, the patient will be excluded at that point. Incapacitated patients that object to participation, will not be included in the study. Incapacitated patients that object during participation, will immediately be excluded at that moment. The patient or, if he/she is incapacitated, the legal representative, can refuse informed consent without any consequences for further treatment and can withdraw informed consent at any time during the study, which is stated in the information letter.

11.4 BENEFITS AND RISK ASSESSMENT, GROUP RELATEDNESS

EEG is a safe, non-invasive and painless procedure that is used regularly in standard medical practice and does not involve electromagnetic radiation. The Waveguard™ dry electrode cap and the eego™ amplifier are both CE marked and have been safely used in several clinical studies (see chapter 6.3). We expect no health risks and no to minimal discomfort (only during measurement) associated with participation. For patients, there is no benefit of participation in the study.

11.5 COMPENSATION FOR INJURY

As we do not expect any risks related to participation in this study, we deem exemption from the requirement to insure cover for damage to research subjects caused by this study applicable.

11.6 INCENTIVES

For the patient, there is no benefit of participation in the study.

12. ADMINISTRATIVE ASPECTS AND MONITORING

12.1 HANDLING AND STORAGE OF DATA AND DOCUMENTS

EEG data within phase 1 and 2 will be stored locally. After an ambulant EEG is performed in phase 3 and 4, the EEG data will be sent to a secure external server, containing only EEG data, date and time of the EEG recording and an assigned patient identification code. These data will be sent from the

secure external server to the coordinating investigator. The paramedic that has performed the EEG will fill out a predesigned form with patient characteristics and patient identification code and send it to the local coordinating investigator per mail. A log with all identification codes and identifying information will be kept digitally by the local coordinating investigator, separate from the study database. This log will be password encrypted and saved on the secured drive system of the participating center, only being accessible to the local research staff. Other study data, as described in chapter 7.3, will in part be retrieved from the ambulance service and from the electronic patient file. All study data, apart from identifying information, imaging data and EEG data, will be stored in a digital database (Castor), also password encrypted and only accessible to the research staff of the participating center and the coordinating hospital. EEG and imaging data will finally be stored on a network drive within the AMC, accessible only to authorized research staff of the coordinating hospital. Handling of data will comply with the General Data Protection Regulation (in Dutch: Algemene Verordening Gegevensbescherming).

12.2 MONITORING AND QUALITY ASSURANCE

Not applicable.

12.3 AMENDMENTS

Amendments are changes made to the research after a favourable opinion by the accredited METC has been given. All amendments will be notified to the METC that gave a favourable opinion.

12.4 ANNUAL PROGRESS REPORT

The investigator will submit a summary of the progress of the study to the accredited METC once a year. Information will be provided on the date of inclusion of the first subject, numbers of subjects included and numbers of subjects that have completed the trial, serious adverse events, amendments and other problems.

12.5 TEMPORARY HALT AND (PREMATURE) END OF STUDY REPORT

The investigator will notify the accredited METC of the end of the study within a period of 8 weeks. The end of the study is defined as the last patient's last visit. The investigator will notify the METC immediately of a temporary halt of the study, including the reason of such an action. In case the study is

ended prematurely, the investigator will notify the accredited METC within 15 days, including the reasons for the premature termination. Within one year after the end of the study, the investigator will submit a final study report with the results of the study, including any publications/abstracts of the study, to the accredited METC.

13. RISK ANALYSIS

EEG is a safe, non-invasive and painless procedure that is used regularly in standard medical practice. It does not involve electromagnetic radiation. We will use CE marked products only for measurements. We expect no health risks and no to minimal discomfort (only during the measurement) associated with participation.

14. REFERENCES

1. Thrift AG, Thayabaranathan T. Global stroke statistics. Int J Stroke. 2017 Jan;12(1):13-32.

2. Vaartjes I, O'Flaherty M, Capewell S, Kappelle J, Bots M. Remarkable decline in ischemic stroke mortality is not matched by changes in incidence. Stroke. 2013;44(3):591-7.

3. Centraal Bureau voor de Statistiek, StatLine (online database).

4. National Institute of Neurological Disorders and Stroke rt-PA Stroke Study Group. Tissue plasminogen activator for acute ischemic stroke. N Engl J Med. 1995 Dec 14;333(24):1581-7.

5. HERMES collaborators. Endovascular thrombectomy after large-vessel ischaemic stroke: a meta-analysis of individual patient data from five randomised trials. Lancet. 2016 Apr 23;387(10029):1723-31.

6. Nogueira RG, Jadhav AP et al. Thrombectomy 6 to 24 Hours after Stroke with a Mismatch between Deficit and Infarct. N Engl J Med. 2018 Jan 4;378(1):11-21.

7. Albers GW, Marks MP et al. Thrombectomy for Stroke at 6 to 16 Hours with Selection by Perfusion Imaging. N Engl J Med. 2018 Feb 22;378(8):708-718.

8. Saver JL, Goyal M et al. Time to Treatment With Endovascular Thrombectomy and Outcomes From Ischemic Stroke: A Meta-analysis. JAMA. 2016 Sep 27;316(12):1279-88.

9. Froehler MT, Saver JL et al. Interhospital Transfer Before Thrombectomy Is Associated With Delayed Treatment and Worse Outcome in the STRATIS Registry (Systematic Evaluation of Patients Treated With Neurothrombectomy Devices for Acute Ischemic Stroke). Circulation. 2017 Dec 12; 136(24): 2311–2321.

10. Turc G, Maïer B et al. Clinical scales do not reliably identify acute ischemic stroke patients with large-artery occlusion. Stroke. 2016;47:1466–1472.

11. Fassbender K, Grotta JC et al. Mobile stroke units for prehospital thrombolysis, triage, and beyond: benefits and challenges. Lancet Neurol. 2017 Mar;16(3):227-237.

12. Finnigan SP, Rose SE et al. Correlation of quantitative EEG in acute ischemic stroke with 30-day NIHSS score: comparison with diffusion and perfusion MRI. Stroke. 2004 Apr;35(4):899-903.

13. Finnigan S, Van Putten MJ et al. EEG in ischaemic stroke: quantitative EEG can uniquely inform (sub-)acute prognoses and clinical management. Clin Neurophysiol. 2013 Jan;124(1):10-9.

14. Van Putten MJ, Tavy DL et al. Continuous Quantative EEG monitoring in hemispheric stroke patients using the brain symmetry index. Stroke. 2004 Nov;35(11):2489-92.

15. Lam AM et al, Manninen PH et al. Monitoring electrophysiologic function during carotid endarterectomy: a comparison of somatosensory evoked potentials and conventional electroencephalogram. Anesthesiology. 1991 Jul;75(1):15-21.

16. Thirumala PD, Thiagarajan K et al. Diagnostic accuracy of EEG changes during carotid endarterectomy in predicting perioperative strokes. J Clin Neurosci. 2016 Mar;25:1-9.

17. Anastasi AA, Falzon O et al. Brain Symmetry Index in healthy and stroke patients for assessment of prognosis. Stroke Res Treat. 2017;2017:8276136.

18. Finnigan S, Wong A et al. Defining abnormal slow EEG activity in acute ischaemic stroke: Delta/alpha ratio as an optimal QEEG index. Clin Neurophysiol. 2016 Feb;127(2):1452-1459.

19. Van Kaam RC, Van Putten MJAM et al. Contralesional Brain Activity in Acute Ischemic Stroke. Cerebrovasc Dis. 2018;45(1-2):85-92.

20. Lopez-Gordo MA, Sanchez Morillo D. Dry EEG Electrodes. Sensors (Basel). 2017 Jul; 14(7): 12847-12870.

21. Hajian-Tilaki K. Sample size estimation in diagnostic test studies of biomedical informatics. J Biomedical Inform. 2014 Apr; 48:193-204.

22. Fiedler P, Pedrosa P et al. Novel Multipin Electrode Cap System for Dry Electroencephalography. Brain Topogr. 2015 Sep;28(5):647-656.

23. Zhongxiang D, De Souza S et al. EEG Cortical Connectivity Analysis of Working Memory Reveals Topological Reorganization in Theta and Alpha Bands. Front Hum Neurosci. 2017; 11:237.

24. Yang Y, Guliyev B et al. Dynamic Causal Modeling of the Cortical Responses to Wrist Pertubations. Front Neurosci. 2017; 11:518.

25. Ehinger BV, Fischer P et al. Kinesthetic and vestibular information modulate alpha activity during spatial navigation: a mobile EEG study. Front Hum Neurosci. 2014; 8:71.

26. Stone DB, Tamburro G et al. Automatic removal of physiological artifacts in EEG: the optimized fingerprint method for sports science applications. Front Hum Neurosci. 2018; 12:96.

27. Barzegaran E, Vildavski VY. Fine structure of posterior alpha rhythm in human EEG: frequency components, their cortical sources and temporal behavior. Sci Rep. 2017; 7:8249.

28. Bashford J, Wickham A et al. Preprocessing surface EMG data removes voluntary muscle activity and enhances SPiQE fasciculation analysis. Clinical Neurophysiology. 2020 Jan; 131(1): 265–273.

29. Tamborska A, Bashford J et al. Non-invasive measurement of fasciculation frequency demonstrates diagnostic accuracy in amyotrophic lateral sclerosis. Brain Communications. 2020 Sep; 2(2): fcaa141.

30. Kamavuako E, Scheme E et al. Determination of optimum threshold values for EMG time domain features; a multi-dataset investigation. J. Neural Eng. 2016 Jun; 13(4): 46011.

31. Rezaie R, Schiller K et al. The Clinical Utility of Transcranial Magnetic Stimulation in Determining Hemispheric Dominance for Language: A Magnetoencephalography Comparison Study. Journal of Clinical Neurophysiology. 2020 Mar; 37 (2): 90-103.

32. Fiedler P, Muhle R et al. IEEE Trans Neural Syst Rehabil Eng. 2018 Apr;26(4):750-757.

33. Roy Y, Banville H et al. Deep learning for electroencephalogram (EEG) classification tasks: a review. J. Neural Eng. 2019 Jun;16(3):031001.

34. Wang P, Jiang A et al. LSTM-Based EEG Classification in Motor Imagery Tasks. IEEE Transactions on Neural Systems and Rehabilitation Engineering. 2018 Nov;26(11):2086-2095.

35. Kumar S, Sharma A et al. Brain wave classification using long shortterm memory network based OPTICAL predictor. Sci Rep. 2019 9:9153.

36. Jansen TC, Kompanje EJ et al. Deferred proxy consent in emergency critical care research: ethically valid and practically feasible. 2009 Jan;37:S65-8.

37. Annane D, Outin H et al. The effect of waiving consent on enrollment in a sepsis trial. Intensive Care Med. 2004 Feb;30(2):321-324.

38. Feldman WB, Kim AS et al. Effect of waivers of consent on recruitment in acute stroke trials. Neurology. 2016 Apr; 86(16):1543-1551.
